# Systemic Candidiasis and TLR2 Agonist Exposure Impact the Antifungal Response of Hematopoietic Stem and Progenitor Cells

**DOI:** 10.3389/fcimb.2018.00309

**Published:** 2018-09-03

**Authors:** Alba Martínez, Cristina Bono, Javier Megías, Alberto Yáñez, Daniel Gozalbo, M. Luisa Gil

**Affiliations:** ^1^Departamento de Microbiología y Ecología, Universitat de València, Burjassot, Spain; ^2^Estructura de Recerca Interdisciplinar en Biotecnologia i Biomedicina, Universitat de València, Burjassot, Spain; ^3^Departamento de Patología, Universitat de València, Valencia, Spain; ^4^Department of Biomedical Sciences, Board of Governors Regenerative Medicine Institute, Cedars-Sinai Medical Center, Los Angeles, CA, United States

**Keywords:** *Candida albicans*, hematopoietic stem and progenitor cells, TLR2, macrophages, innate immunity, host-pathogen interactions

## Abstract

We have previously demonstrated that *Candida albicans* induces differentiation of hematopoietic stem and progenitor cells (HSPCs) toward the myeloid lineage both *in vitro* and *in vivo* in a TLR2- and Dectin-1-dependent manner, giving rise to functional macrophages. In this work, we used an *ex vivo* model to investigate the functional consequences for macrophages derived from HSPCs *in vivo*-exposed to Pam_3_CSK_4_ (a TLR2 agonist) or *C. albicans* infection. Short *in vivo* treatment of mice with Pam_3_CSK_4_ results in a tolerized phenotype of *ex vivo* HSPC-derived macrophages, whereas an extended Pam_3_CSK_4_ treatment confers a trained phenotype. Early during candidiasis, HSPCs give rise to macrophages trained in their response to Pam_3_CSK_4_ and with an increased fungicidal activity; however, as the infection progresses to higher fungal burden, HSPC-derived macrophages become tolerized, while their fungicidal capacity is maintained. These results demonstrate that memory-like innate immune responses, already described for monocytes and macrophages, also take place in HSPCs. Interestingly, extended Pam_3_CSK_4_ treatment leads to an expansion of spleen HSPCs and myeloid cells, and drastically reduces the fungal burden in the kidney and spleen during systemic *C. albicans* infection. This protection against tissue invasion is abrogated by immunodepletion of HSPCs, suggesting their protective role against infection in this model. In addition, HSPCs produce *in vitro* cytokines and chemokines in response to *C. albicans* and Pam_3_CSK_4_, and these secretomes are capable of inducing myeloid differentiation of HSPCs and modulating peritoneal macrophage cytokine responses. Taken together, these data assign an active role for HSPCs in sensing pathogens during infection and in contributing to host protection by diverse mechanisms.

## Introduction

*Candida albicans* is the microorganism most frequently causing opportunistic fungal infections. Systemic candidiasis are life-threatening infections whose frequency has increased as a result of an expanding hospitalized and immunocompromised population. Phagocytes, such as neutrophils, dendritic cells, monocytes and macrophages, are crucial for resistance to candidiasis. During infection, these myeloid cells detect the microorganisms and microbial components by using pattern recognition receptors (PRRs), and are responsible for microbial killing, antigen processing and presentation to initiate the adaptive immune response, as well as for releasing pro-inflammatory cytokines and chemokines to recruit and activate other leukocytes. *C. albicans* cells are sensed directly by myeloid cells through many PRRs including different members of the Toll-like receptor (TLR) and C-type lectin receptor (CLR) families (Luisa Gil et al., 2016; Lionakis and Levitz, 2017).

It has been known for a decade that, in addition to mature myeloid cells, hematopoietic stem and progenitor cells (HSPCs) also express some functional PRRs. TLR signaling on hematopoietic stem cells (HSCs) provokes cell cycle entry and myeloid differentiation (Nagai et al., 2006; Sioud et al., 2006; De Luca et al., 2009). This observation opened new perspectives on host-pathogen interactions concerning mechanisms responsible for emergency myelopoiesis during infection (Scumpia et al., 2010; King and Goodell, 2011; Yáñez et al., 2013a; Boettcher and Manz, 2017).

Our group has previously demonstrated that *C. albicans* induces proliferation of HSPCs and their differentiation toward the myeloid lineage both *in vitro* and *in vivo* (Yáñez et al., 2009, 2010, 2011; Megías et al., 2012, 2013). This response requires signaling through TLR2 and Dectin-1, and gives rise to functional macrophages that are able to internalize yeasts and secrete proinflammatory cytokines. These preliminary results indicated that self-/non-self-discrimination also occurs at the level of HSPCs, where PRR-mediated signaling may lead to reprogramming early progenitors to rapidly replenish the innate immune system and generate the most necessary mature cells to deal with the pathogen.

Moreover, using an *in vitro* model of HSPC differentiation, we have shown that detection of pathogen-associated molecular patterns (PAMPs) by HSPCs impacts the antimicrobial function of the macrophages they produce (Yáñez et al., 2013b). Pure soluble TLR2 and TLR4 ligands generate macrophages with a diminished ability to produce inflammatory cytokines (tolerized macrophages), whereas HSPC activation in response to *C. albicans* leads to the generation of macrophages that produce higher levels of cytokines (trained macrophages) than control M-CSF-derived macrophages (Megías et al., 2016). In fact, the ability of macrophages to produce inflammatory cytokines is extremely dependent on how the HSPCs from which they are derived receive and integrate multiple microenvironmental signals; the tolerized or trained phenotype depends on the combination of signals they receive (PRRs and CSFs), as well as on the timing of the HSPC activation by the different stimuli (Martínez et al., 2017).

Recent studies have challenged the dogma that adaptive immunity is the only arm of the immune response with memory, demonstrating that innate immune cells (especially monocytes and macrophages) can display some memory characteristics (Goodridge et al., 2016; Netea et al., 2016). After first priming, the alteration of the innate immune system would be such that upon re-exposure to the same or heterologous stimuli, it would display a trained or tolerized response. For example, exposure of monocytes or macrophages to *C. albicans* enhances their subsequent response to stimulation (trained immunity), while TLR2 and TLR4 ligands confer a long-lasting reduced inflammatory cytokine production (tolerance) to macrophages. Therefore, our previous *in vitro* data (Yáñez et al., 2013b; Megías et al., 2016; Martínez et al., 2017) indicate that this concept of “innate immune memory” may apply not only to differentiated cells but also to HSPCs. Supporting this idea, it has been recently shown that intravenous vaccination with Bacillus Calmette-Guérin “educates” HSCs to generate trained monocytes/macrophages that protect mice against tuberculosis (Kaufmann et al., 2018).

Here, we extend our previous *in vitro* studies to an *ex vivo* model in order to demonstrate that systemic candidiasis or TLR2 agonist exposure *in vivo* impacts the antifungal phenotype of the macrophages produced *in vitro* from purified HSPCs. Moreover, sustained systemic exposure to a TLR2 agonist protects mice from systemic *C. albicans* infection, and leads to an expansion of spleen HSPCs and myeloid cells; this protection is abrogated by immunodepletion of expanded HSPCs, indicating their protective role against infection. In addition, HSPCs secrete several cytokines and chemokines in response to *C. albicans* and a TLR2 agonist (HSPC secretomes). These secretomes are capable of inducing HSPCs to differentiate into myeloid cells and modulating cytokine production by peritoneal macrophages. Taken together, these data indicate that HSPCs can sense pathogens during infection and contribute to protect the host by different mechanisms.

## Materials and methods

### Mice

TLR2^−/−^ mice (C57BL/6 background) provided by Dr. Shizuo Akira (Osaka University, Osaka, Japan) were bred and maintained at the animal production service facilities (SCSIE, University of Valencia); wild-type C57BL/6 mice were purchased from Harlan Ibérica (Barcelona, Spain). Mice of both sexes between 8 and 12 weeks old were used. This study was carried out in strict accordance with the “Real Decreto 1201/2005, BOE 252” for the Care and Use of Laboratory Animals of the “Ministerio de la Presidencia,” Spain. All efforts were made to minimize suffering. The protocols were approved by the Committee on the Ethics of Animal Experiments of the University of Valencia, Generalitat Valenciana (Permit Numbers: 2017/VSC/PEA/00004; 00024; 00084).

### Yeasts strains and preparation of fungal cells

Starved and inactivated *C. albicans* ATCC 26555 yeasts were prepared as previously described (Villamón et al., 2004; Martínez et al., 2017). Briefly, cells of *C. albicans* strain ATCC 26555 were grown in YPD medium (1% yeast extract, 2% peptone, 2% glucose) at 28°C up to the late exponential growth phase (A_600nm_ 0.6-1), collected and washed with water. Cells were resuspended in water, and maintained for 3 h at 28°C with shaking, and afterwards at 4°C during 48 h (starved yeast cells). Starved yeast cells were inoculated (200 μg dry weight of cells per ml) in a minimal synthetic medium, and incubated for 3 h at 28°C. For inactivation, yeast cells were resuspended (20 × 10^6^ cells/ml) in 4% paraformaldehyde (fixation buffer, eBioscience, San Diego, CA) and incubated for 1 h at room temperature. After treatment, fungal cells were extensively washed in PBS and brought to the desired cell density in cell culture medium.

Yeast cells of *C. albicans* PCA2, a low-virulence non-germinative strain, were grown in YPD medium at 28°C up to the late exponential growth phase, collected, washed in PBS, and brought to the desired cell density in complete cell culture medium (see below). All procedures were performed under conditions designed to minimize endotoxin contamination as described elsewhere (Villamón et al., 2004).

### *C. albicans* infection model

WT control or Pam_3_CSK_4_-treated mice were injected intraperitoneally with 45 × 10^6^ or 30 × 10^6^ starved yeasts of *C. albicans* ATCC 26555 in 200 μl of PBS. To assess the tissue outgrowth of the microorganism, the fungal burden in the kidney and spleen at different days post-infection was determined. The organs were weighed, homogenized in 1 ml of PBS and dilutions of the homogenates were plated on Sabouraud dextrose agar. The colony forming units (CFUs) were counted after 24 h of incubation at 37°C, and expressed as CFUs per gram of tissue.

To study the effect of c-Kit-progenitor-cell depletion, 500 μg of the anti-c-Kit antibody ACK2 (eBioscence) or isotype control (rat IgG_2b_, clone eB149/10H5, from eBioscence) were given to mice intraperitoneally 2 days before infection.

### Isolation of splenocytes and peritoneal macrophages

Splenocytes were obtained from control or Pam_3_CSK_4_-treated mice as described elsewhere (Murciano et al., 2007; Yáñez et al., 2011). Briefly, total spleen cells were obtained by collagenase D treatment of the organ, erythrocytes were lysed using ammonium chloride buffer (BD FACS™ lysing solution), and cells were washed and counted.

Resident peritoneal macrophages were harvested by instilling and withdrawing 10 ml of complete cell culture medium: RPMI 1640 medium supplemented with 2 mM L-glutamine, 5% heat-inactivated fetal bovine serum, and 1% penicillin-streptomycin stock solution (Gibco, Barcelona, Spain). Cells were washed once with the same medium, and plated at a density of 1.5 x 10^5^ cells in 200 μl of medium per well, in a 96 well tissue culture plate. Peritoneal macrophages were allowed to adhere for 5 h at 37°C in a 5 % CO_2_ atmosphere, the non-adherent cells were removed, and the adherent macrophages were cultured for 72 h prior to be challenged with the indicated stimuli.

### Purification of Lin^−^ cells and *ex vivo* differentiation

Lin^−^ cells were purified as previously described (Yáñez et al., 2011; Megías et al., 2012). Briefly, murine bone marrow (obtained by flushing the femurs and tibias) and spleen (obtained as above explained) were depleted of lineage-positive cells by immunomagnetic cell sorting (negative selection) using MicroBeads and a cocktail of antibodies against a panel of lineage antigens [CD5, CD45R (B220), CD11b, Gr-1 (Ly-6G/C), 7-4, and Ter-119] according to the manufacturer's instructions (Miltenyi Biotec, Madrid, Spain).

Purified cells were immediately cultured in complete cell culture medium supplemented with two cytokines: 20 ng/ml of stem cell factor (SCF, Peprotech, Rocky Hill, NJ) to support the survival of HSPCs, and 50 ng/ml of macrophage-colony-stimulating factor (M-CSF, Miltenyi Biotec, Madrid, Spain) to induce their differentiation to macrophages. Where indicated, cultures also contained inactivated yeasts of *C. albicans* (1:7.5 murine cell:yeast ratio) as stimuli throughout the 7 days of culture. Adherent cells were harvested at day 7.

### Obtaining of secretomes produced by Lin^−^ cells

Lin^−^ cells were cultured in a serum-free medium (StemPro-34 SFM medium, Gibco, Barcelona, Spain), containing 2 mM L-glutamine, 1% penicillin-streptomycin stock solution, and two cytokines: 20 ng/ml of SCF and 100 ng/ml of Flt3 ligand (FL, Peprotech, Rocky Hill, NJ). Cells were cultured in 96-well plates at a density of 50,000 cells in 250 μl, and challenged for 72 h with the indicated stimuli [1 μg/ml of Pam_3_CSK_4_ or inactivated *C. albicans* yeasts (1:5 murine cell:yeast ratio)]. Then, secretomes (conditioned media) were collected and used for the different assays. The effect of secretomes from HSPCs stimulated with Pam_3_CSK_4_ was determined on HSPCs or peritoneal macrophages from TLR2^−/−^ mice.

### Measurement of cytokine production

M-CSF macrophages were plated in 96-well plates at a density of 50,000 cells in 200 μl of complete cell culture medium. Splenocytes were plated in 24-well plates at a density of 2.5 × 10^6^ cells in 0.5 ml of complete cell culture medium. Cells were challenged with the indicated stimuli for 24 h and cell-free supernatants were then harvested and tested for TNF-α release using a commercial ELISA kit (eBioscience, San Diego, CA). The stimuli used were Pam_3_CSK_4_ (100 ng/ml), Ultrapure *Escherichia coli* LPS (100 ng/ml) (Invivogen, San Diego, CA), and inactivated yeasts of *C. albicans* (2 x 10^6^ yeasts/ml). Triplicate samples were analyzed in each assay.

TNF-α and IL-6 levels in the secretomes produced by HSPCs were measured using commercial ELISA kits (eBioscience, San Diego, CA). Moreover, levels of 40 cytokines were determined in the secretomes using a mouse cytokine array (RayBio Mouse inflammation antibody array C1) according to the manufacturer's instructions (RayBiotech, Norcross, GA).

### *C. albicans* killing assay

The assay was performed with M-CSF-derived macrophages or with splenocytes. Macrophages were plated in 96-well plates at a density of 200,000 cells in 150 μl of complete cell culture medium. Macrophages were challenged with viable PCA2 yeasts at a 1:3 ratio (murine cell:yeast), settled onto the macrophages by centrifugation, and incubated for 1 h. As a control, *C. albicans* cells were inoculated in culture medium without murine cells. Splenocytes were plated in 24-well plates at a density of 2.5 × 10^6^ cells in 0.5 ml of complete cell culture medium. Splenocytes were challenged with 100,000 viable PCA2 yeasts settled onto the cells by centrifugation, and incubated for 4 h. As a control, *C. albicans* cells were inoculated in culture medium without murine cells.

After co-incubation, samples were diluted in water, plated on Sabouraud dextrose agar, and incubated overnight at 37°C to determine CFUs. Colonies were counted, and killing percentages were determined as follows: % killing = [1 – (CFUs sample at *t* = 1 or 4 h)/(CFUs control at *t* = 1 or 4 h)] × 100. A non-germinative strain (PCA2) was chosen for killing assays in order to facilitate determination of CFUs after the incubation period, as no germ tube (hyphae) aggregates are formed. Triplicate samples were analyzed in each assay.

### Antibodies and flow cytometry analyses

Cell suspensions were labeled with various combinations of antibodies, and analyzed by flow cytometry. The following antibodies were used in this study: PE-labeled anti-CD11b (clone M1/70 from eBioscience), PerCP-Cy5.5-labeled anti-MHC class II (clone M5/114.15.2 from BD Pharmigen), APC-Cy7-labeled anti-Ly6C (clone AL-21 from BD Pharmigen), BUV395-labeled anti-Ly6G (clone AL-21 from BD Pharmigen), APC-labeled anti-CD11c (clone N418 from Miltenyi Biotec), cocktail of biotinylated anti-lineage antigens [CD5, CD45R (B220), CD11b, Gr-1 (Ly-6G/C), 7-4, and Ter-119], and PE-labeled anti-biotin (clone REA746) both from Miltenyi Biotec, and PE-Vio770-labeled anti-c-Kit (clone 3C11 from Miltenyi Biotec).

Flow cytometry analyses were performed on a LSR Fortessa cytometer (BD Biosciences), and data were analyzed with FACSDiva and FlowJo 10 software.

### Statistical analysis

Statistical differences were determined using one-way ANOVA followed by Dunnett's test for multiple comparisons and two-tailed Student's *t*-test for dual comparisons. Data are expressed as mean ± SD. Significance was accepted at ^*^P < 0.05, ^**^ *P* < 0.01, ^***^ *P* < 0.001, and ^****^ *P* < 0.0001 levels.

## Results

### *C. albicans* infection impacts the cytokine production and the fungicidal activity of the *ex vivo* produced macrophages

**Figure 1:**
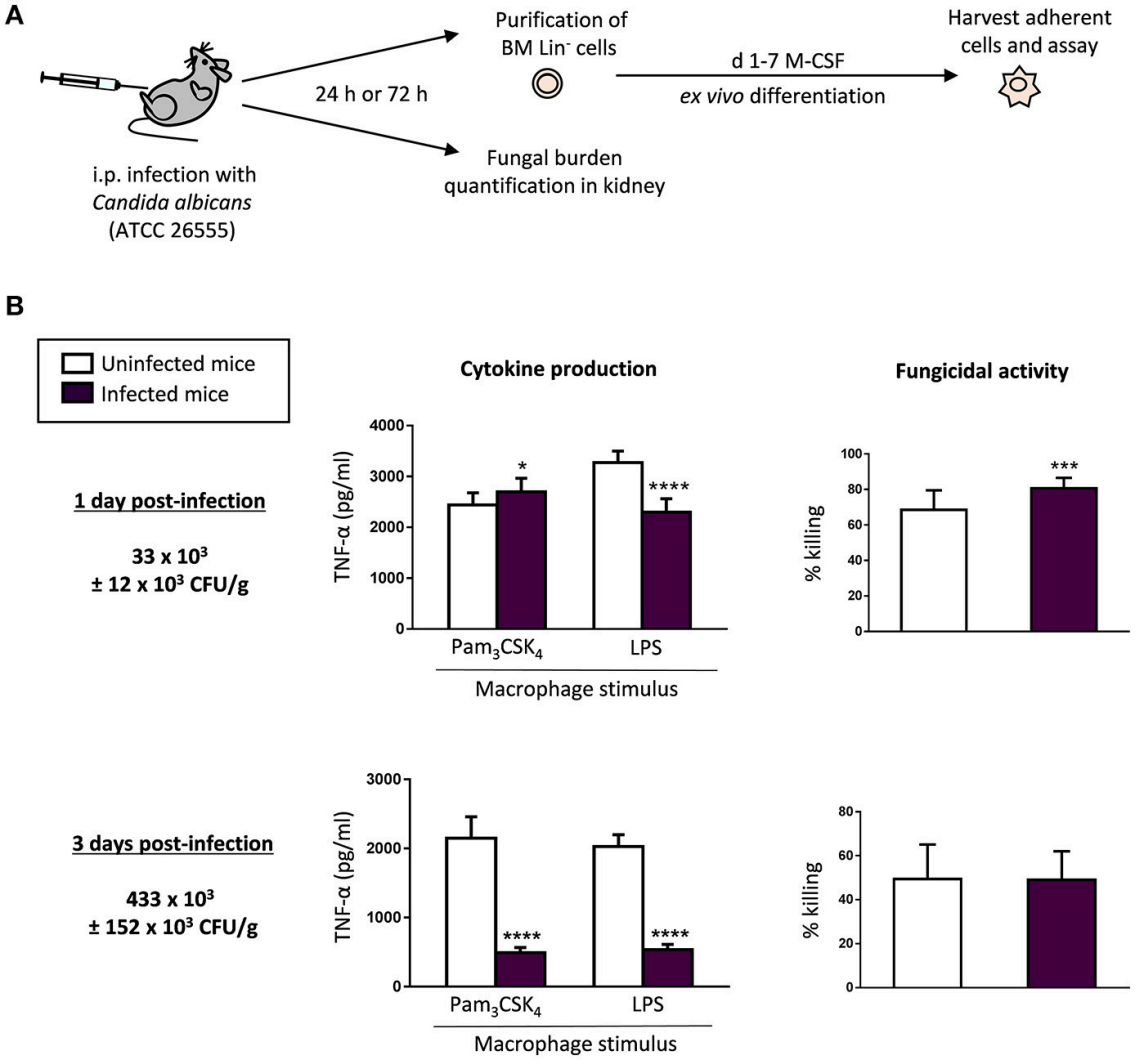
*C. albicans* infection impacts the cytokine production and the fungicidal activity of the *ex vivo* produced macrophages. **(A)** Schematic protocol (as described in section Materials and Methods). WT mice were injected intraperitoneally with 45 × 10^6^ yeasts of *C. albicans* ATCC 26555. 1 or 3 days post-infection mice were sacrificed to assess the outgrowth of the yeasts in the kidney, and to isolate the bone marrow. Lin^−^ HSPCs were purified from bone marrow, plated at a density of 200,000 cells in 4 ml of culture medium containing SCF, M-CSF and amphotericin B, and incubated for 7 days to induce macrophage differentiation. **(B)** The fungal burden in the kidneys is expressed as CFUs per gram of tissue. For cytokine assays, macrophages were plated at a density of 50,000 cells in 200 μl of complete cell culture medium and challenged with Pam_3_CSK_4_ (100 ng/ml) or LPS (100 ng/ml) for 24 h. TNF-α levels in cell-free culture supernatants were measured by ELISA. For fungicidal activity determination, macrophages were plated at a density of 200,000 cells in 200 μl of complete cell culture medium and challenged with viable PCA2 yeasts at a 1:3 ratio (murine cell:yeast) for 1 h. *C. albicans* cells were also inoculated in culture medium without murine cells (control). After incubation, samples were diluted, plated on Sabouraud dextrose agar and incubated overnight at 37°C; CFUs were counted and killing percentages were determined as follows: % killing = [1 – (CFUs sample at *t* = 1 h)/(CFUs control at *t* = 1 h)] × 100. Triplicate samples were analyzed in each assay. Results are expressed as means ± SD of pooled data from two experiments. *P < 0.05, ***P < 0.001, and ****P < 0.0001 with respect to macrophages derived from control uninfected mice.

We have previously reported that *in vitro* detection of TLR2 and Dectin-1 ligands (including inactivated yeasts of *C. albicans*) by HSPCs impacts the antimicrobial function of the macrophages they produce (Yáñez et al., 2013b; Megías et al., 2016; Martínez et al., 2017). The observed *in vitro* effect of *C. albicans* on HSPCs may be of biological relevance *in vivo* during infection. Therefore, we now sought to determine whether HSPCs may sense microorganisms *in vivo* using a mouse model of systemic candidiasis, and whether this may alter the function of the macrophages they produce *ex vivo*. C57BL/6 mice were infected via intraperitoneal injection of a virulent strain of *C. albicans* (45 × 10^6^ yeasts per mouse). At days 1 or 3 post-infection, Lin^−^ cells were purified from mice bone marrow and differentiated into macrophages in M-CSF cultures in the presence of 2.5 μg/ml of amphotericin B to prevent potential fungal growth. To assess the tissue outgrowth of the microorganism in infected mice, the fungal burden was determined in the kidney, the target organ in this invasive model of candidiasis (Figure 1A). The dose of yeasts injected resulted in a low and high number of CFUs at days 1 and 3, respectively. The *ex vivo* differentiated macrophages were counted and plated at equal cell numbers for stimulation with TLR agonists to assess their ability to produce TNF-α. Unstimulated macrophages served as negative controls. Cytokine production in response to Pam_3_CSK_4_ was significantly increased in macrophages generated from low-infected mice (day 1 post-infection) compared to macrophages generated from control uninfected mice, whereas macrophages generated from high-infected animals (day 3 post-infection) produced lower cytokine levels than control macrophages. TNF-α production in response to LPS was significantly diminished in macrophages generated from both low- and high-infected mice compared to control macrophages (Figure 1B).

To further characterize the antifungal function of Lin^−^-derived macrophages generated from infected animals, we measured the ability of differentiated cells to kill yeast cells *in vitro*. Macrophages were challenged with viable yeasts at a 1:3 ratio (murine cell:yeast) for 1 h, in order to determine their fungicidal activity. In these conditions, control macrophages were able to kill a significant percentage of yeasts, similar to macrophages obtained from high-infected mice. However, macrophages differentiated from HSPCs purified from low-infected animals possessed an increased fungicidal activity (roughly 80% as compared with 65% of killing by control macrophages; i.e., 23% greater than control macrophages) (Figure 1B).

Therefore, early during the infection, with low fungal burden levels, HSPCs give rise to macrophages trained in response to Pam_3_CSK_4_ and with higher fungicidal activity. Interestingly, when the infection reaches high fungal burden levels, Lin^−^-derived macrophages become tolerized, as they have a diminished ability to produce TNF-α, whereas they keep up their fungicidal capacity. These data collectively indicate that HSPCs sense the infection *in vivo* and this profoundly alters the functional phenotype of the macrophages *ex vivo* derived from them.

### TLR2 agonist treatment *in vivo* impacts the cytokine production and the fungicidal activity of the *ex vivo* produced macrophages

**Figure 2:**
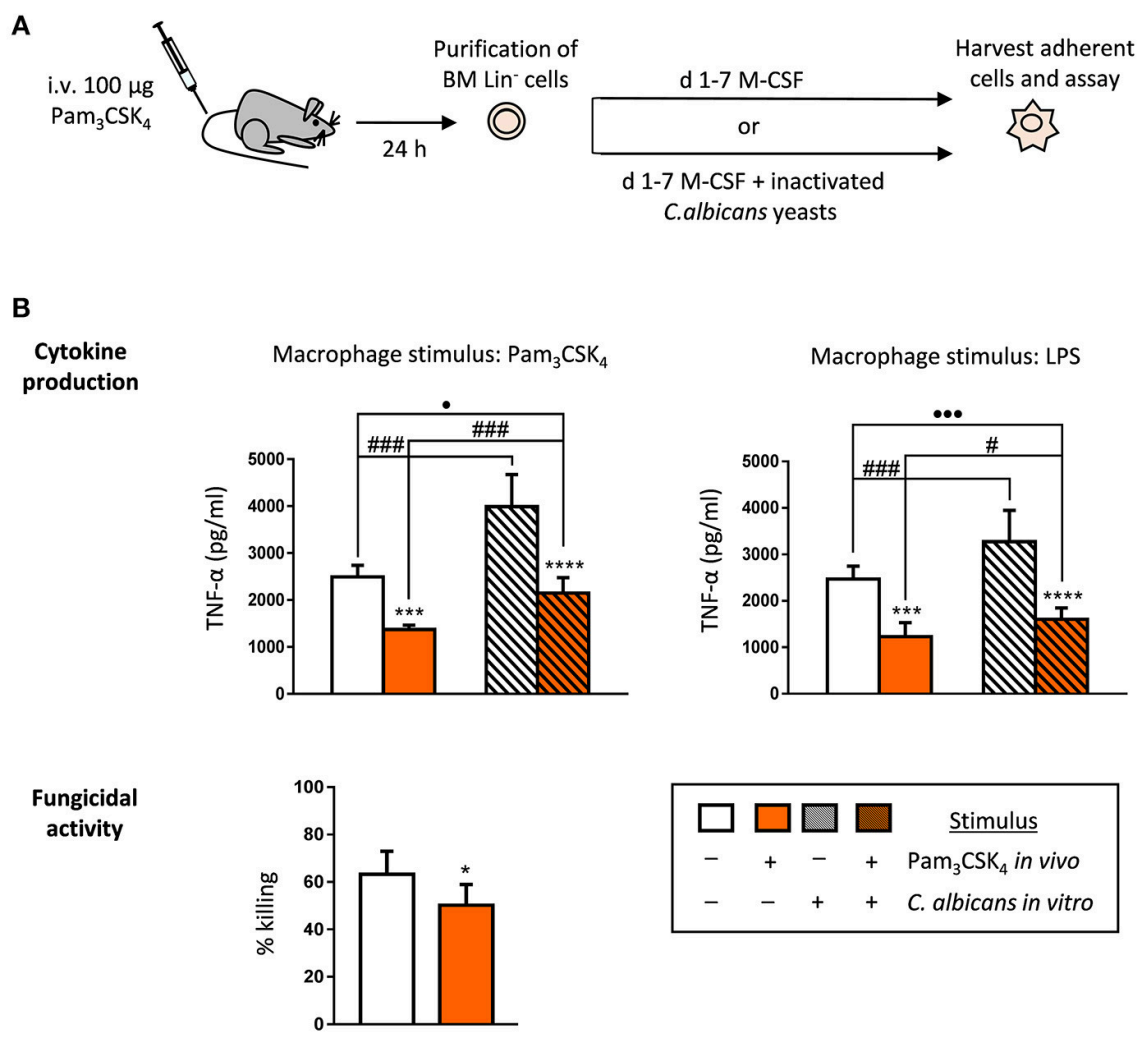
TLR2 agonist short treatment impacts the cytokine production and the fungicidal activity of the *ex vivo* produced macrophages. **(A)** Schematic protocol (as described in section Materials and Methods). WT mice were injected intravenously with 100 μg of Pam_3_CSK_4_ and Lin^−^ HSPCs were recovered from bone marrow on day 1 for *ex vivo* differentiation to macrophages. Lin^−^ cells were plated at a density of 200,000 cells in 4 ml of media containing SCF and M-CSF, and incubated for 7 days to induce macrophage production, in the presence or absence of inactivated yeasts of *C. albicans* (1:7.5 murine cell:yeast ratio). **(B)** For cytokine assays and fungicidal activity determination macrophages were plated and challenged as indicated in Figure 1B. Triplicate samples were analyzed in each assay. Results are expressed as means ± SD of pooled data from two experiments. *P < 0.05, ***P < 0.001, and ****P < 0.0001 with respect to macrophages derived from control untreated mice, ^#^*P* < 0.05 and ^###^*P* < 0.001 with respect to cytokine production by macrophages derived from HSPCs differentiated with M-CSF only, in the absence of inactivated yeasts, and ^•^*P* < 0.05, and ^•••^*P* < 0.001 with respect to macrophages derived from control untreated mice.

Next, we wondered whether *in vivo* exposure of HSPCs to Pam_3_CSK_4_ may alter the function of the macrophages they produce *ex vivo*. First, we used a short treatment model by intravenously injecting mice with one dose of the ligand. 24 h later, Lin^−^ cells were purified from the bone marrow, and cultured with M-CSF for macrophage differentiation in the presence or absence of inactivated *C. albicans* yeasts (Figure 2A). The harvested macrophages were then plated for stimulation with TLR agonists to assess their ability to produce TNF-α. The production of TNF-α in response to Pam_3_CSK_4_ or LPS was significantly diminished in macrophages generated from HSPCs exposed to the TLR2 ligand *in vivo* (tolerized macrophages), compared to control macrophages (differentiated from HSPCs purified from control mice) (Figure 2B, plain white and colored bars). As we have previously reported, *in vitro* HSPC differentiation in the presence of *C. albicans* leads to the generation of macrophages that produce higher levels of TNF-α (trained macrophages) (Figure 2B, grated white bars). Interestingly, differentiation of Lin^−^ cells (purified from Pam_3_CSK_4_-treated mice) in the presence of fungal stimuli partially reversed the tolerized phenotype (Figure 2B, grated colored bars), as macrophages produced higher cytokine levels than *in vivo* Pam_3_CSK_4_ tolerized macrophages (Figure 2B, plain colored bars) but still significantly minor amounts than control macrophages (Figure 2B, plain white bars). In addition, macrophages were challenged with viable yeasts in order to determine their fungicidal activity. Results show that the tolerized macrophages, generated from HSPCs exposed to the TLR2 ligand *in vivo*, have a diminished ability to kill yeast cells *in vitro*, as compared with control macrophages (Figure 2B). Therefore, a short *in vivo* exposure to a TLR2 agonist results in tolerized M-CSF-derived macrophages that can still respond to *in vitro* training by *C. albicans* yeasts, but that have decreased their fungicidal activity.

**Figure 3:**
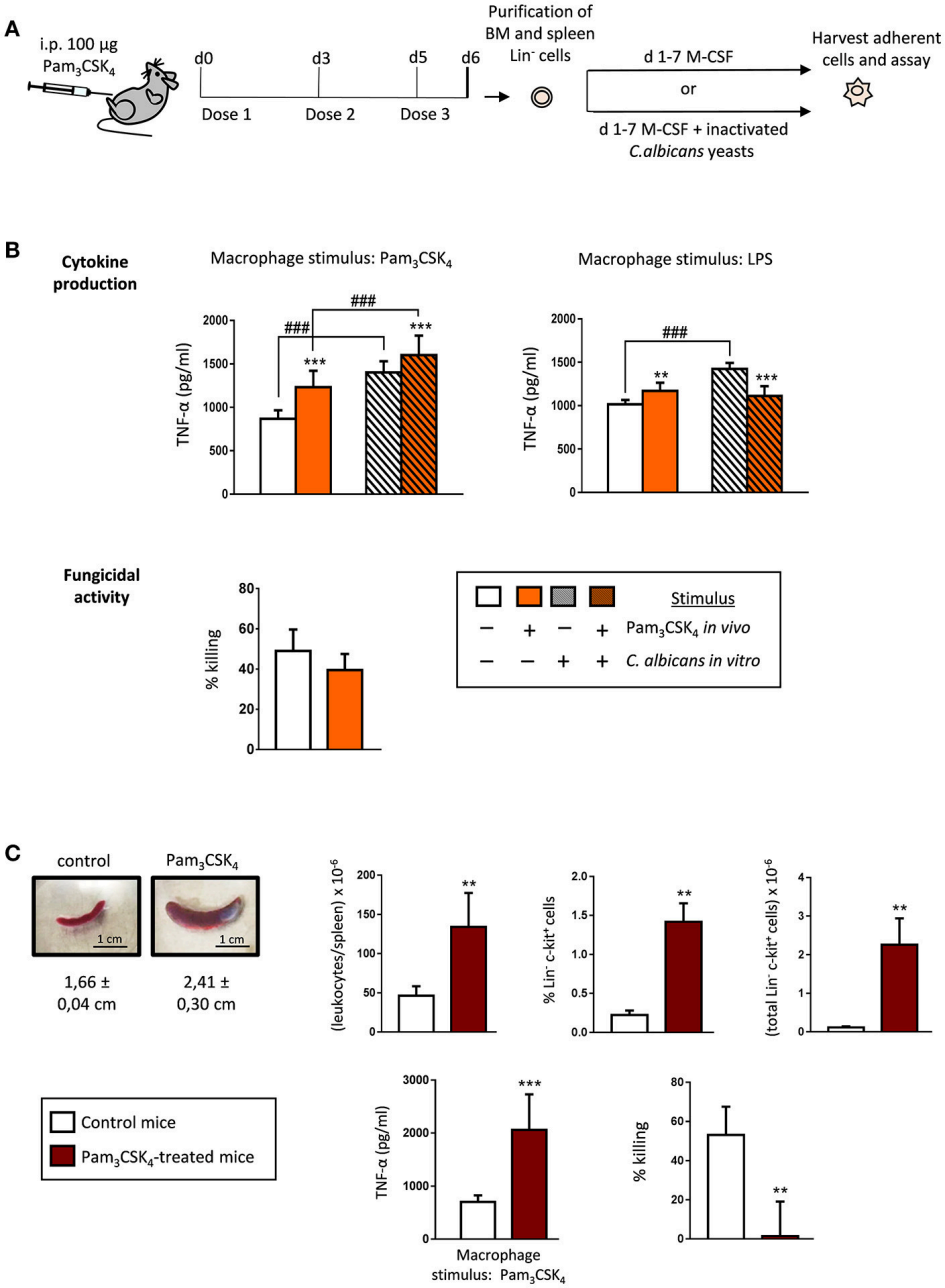
Extended TLR2 agonist treatment impacts the cytokine production and the fungicidal activity of the *ex vivo* produced macrophages derived from bone marrow and spleen HSPCs. **(A)** Schematic protocol (as described in section Materials and Methods). WT mice were given 100 μg of Pam_3_CSK_4_ by intraperitoneal injection at days 0, 3, and 5 (3 doses), and 24 h after the final dose, mice were sacrificed to isolate the bone marrow and the spleen. Lin^−^ HSPCs purified from bone marrow and spleen were plated at a density of 200,000 cells in 4 ml of culture medium containing SCF and M-CSF, and incubated for 7 days to induce macrophage production, in the presence or absence of inactivated yeasts of *C. albicans* (1:7.5 murine cell:yeast ratio). **(B)** Cytokine production and fungicidal activity of macrophages derived from bone marrow HSPCs. Macrophages were plated and challenged as indicated in Figure 1. **(C)** Appearance and size of representative spleens from control (left) and treated (right) mice. Total leukocytes per spleen, and frequency and absolute numbers of Lin^−^ and c-Kit^+^ cells in the spleens, as determined by flow cytometry (*n* = 2 mice per treatment group). TNF-α production and fungicidal activity of macrophages derived from spleen HSPCs (macrophages were plated and challenged as indicated in Figure 1B). Triplicate samples were analyzed in each assay. Results are expressed as means ± SD of pooled data from two experiments. **P < 0.01, and ***P < 0.001 with respect to control macrophages derived from control untreated mice, and ^###^*P* < 0.001 with respect to cytokine production by macrophages derived from HSPCs differentiated with M-CSF only, in the absence of inactivated yeasts.

These findings prompted us to investigate whether this macrophage phenotype, induced by a short systemic Pam_3_CSK_4_ exposure, may also occur after an extended TLR2 agonist treatment. For this purpose, we used a model previously described by Herman et al. (2016), for our *ex vivo* assays. C57BL/6 mice were given 100 μg of Pam_3_CSK_4_ by intraperitoneal injection at days 0, 3 and 5. One day (24 h) after the final dose (day 6) Lin^−^ cells were purified from the bone marrow and differentiated into macrophages with M-CSF, in the presence or absence of inactivated yeasts of *C. albicans* (Figure 3A). In this scenario of extended treatment with the TLR2 ligand, TNF-α production was significantly higher in response to Pam_3_CSK_4_ or LPS, compared to control macrophages (generated from HSPCs from control animals) (Figure 3B, plain white and colored bars). These trained macrophages, generated from HSPCs exposed to the TLR2 ligand *in vivo*, showed no significant differences in their ability to kill yeast cells *in vitro*, as compared with control macrophages (Figure 3B, plain white and colored bars). The presence of fungal cells during differentiation further increased the trained TNF-α response to Pam_3_CSK_4_, but not to LPS stimulation (Figure 3B, grated colored bars). Therefore, after an extended *in vivo* TLR2 agonist exposure, HSPCs give rise to trained M-CSF-derived macrophages, with a similar fungicidal activity than control macrophages.

As previously described by Herman et al. (2016), we observed that the spleens of treated mice were significantly enlarged and had a marked increase in leukocytes and HSPCs; the percentage and the total number of Lin^−^ c-Kit^+^ cells were significantly increased compared with control mice (Figure 3C, Supplementary Figure 1). Lin^−^ cells were also purified from spleen, incubated with M-CSF to induce macrophage differentiation, and cytokine production and fungicidal activity were assessed as indicated above. Macrophages differentiated from bone marrow HSPCs from control mice were used as controls since very few c-Kit^+^ cells were found in control spleens (Figure 3C, Supplementary Figure 1). The production of TNF-α in response to Pam_3_CSK_4_ was significantly higher in macrophages generated from spleen HSPCs exposed to the TLR2 ligand *in vivo*, compared to control macrophages. However, these trained macrophages, generated from spleen HSPCs exposed to the TLR2 ligand *in vivo*, were almost unable to kill yeasts *in vitro* (Figure 3C).

Our data indicate that HSPCs sense the TLR2 agonist *in vivo*, and that this profoundly alters the functional phenotype of the macrophages *ex vivo* derived from them. The ability of HSPC-derived macrophages to produce inflammatory cytokines is dependent on the extent of HSPC exposure to Pam_3_CSK_4_ challenge, as short exposure (one dose) generates tolerized macrophages, while an extended treatment (3 doses) confers a trained phenotype. Moreover, the fungicidal activity is lower for short exposure or similar for extended treatment, compared to control macrophages. Interestingly, spleen HSPCs give raise to macrophages with a very low fungicidal activity, although they show a trained cytokine response.

### Extended TLR2 agonist treatment induces myeloid cell expansion in the spleen, and impacts the cytokine production and the fungicidal activity of splenocytes

**Figure 4:**
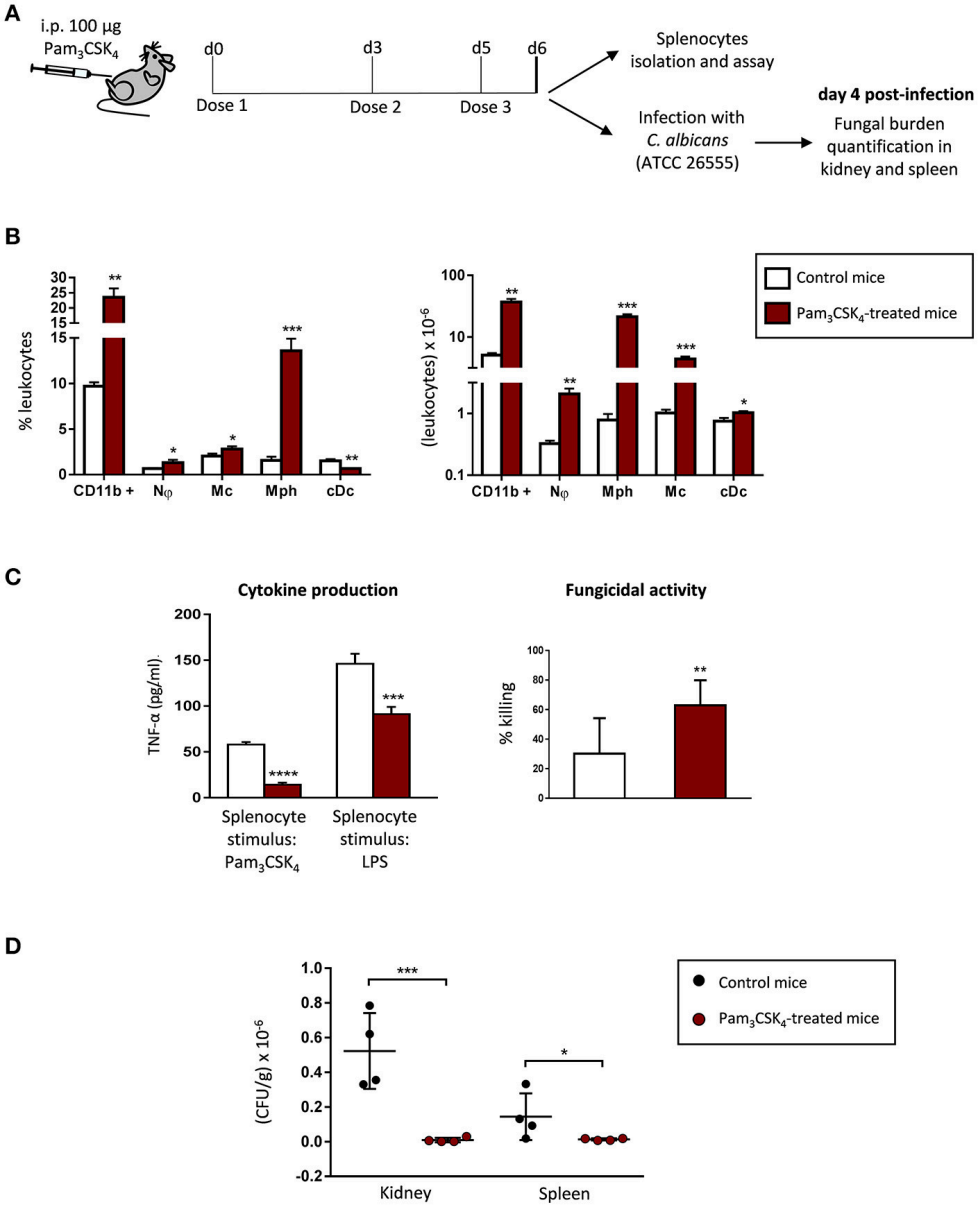
Extended TLR2 agonist treatment protects mice from systemic *C. albicans* infection, induces myeloid cell expansion in the spleen, and impacts the cytokine production and the fungicidal activity of splenocytes. **(A)** Schematic protocol (as described in section Materials and Methods). WT mice were given 100 μg of Pam_3_CSK_4_ by intraperitoneal injection at days 0, 3, and 5 (3 doses), and 24 h after the final dose, mice were injected intraperitoneally with 30 × 10^6^ yeasts of *C. albicans* ATCC 26555, or were sacrificed to isolate the spleen. Four days post-infection, mice were sacrificed to assess the outgrowth of the yeasts in the kidney and spleen. **(B)** Frequency and absolute numbers of myeloid cells per spleen as determined by flow cytometry (*n* = 2 mice per treatment group). Results are expressed as means ± SD of pooled data from two experiments. *P < 0.05, **P < 0.01 and ***P < 0.001 with respect to control mice. Nϕ, neutrophils; Mc, monocytes; Mph, macrophages; cDc, classical dentritic cells. **(C)** TNF-α production and fungicidal activity of splenocytes. Splenocytes were plated at a density of 2.5 × 10^6^ cells in 0.5 ml of complete cell culture medium. For cytokine assays, splenocytes were challenged with Pam_3_CSK_4_ (100 ng/ml) or LPS (100 ng/ml) for 24 h. TNF-α levels in cell-free culture supernatants were measured by ELISA. For fungicidal activity determination, splenocytes were challenged with 100,000 viable PCA2 yeasts and incubated for 4 h. *C. albicans* cells were also inoculated in culture medium without murine cells (control). After incubation, samples were diluted, plated on Sabouraud dextrose agar and incubated overnight at 37°C; CFUs were counted and killing percentages were determined as follows: % killing = [1 – (CFUs sample at *t* = 4 h)/(CFUs control at *t* = 4 h)] × 100. Triplicate samples were analyzed in each assay. Results are expressed as means ± SD of pooled data from two experiments. **P < 0.01, ***P < 0.001 and ****P < 0.0001 with respect to control cells (splenocytes from control mice). **(D)** The fungal burden in kidneys and spleens are expressed as CFUs per gram of tissue. Results are expressed as means ± SD of pooled data from two experiments (*n* = 2 mice each group per experiment). *P < 0.05 and ***P < 0.001 with respect to control mice (infected and not treated with the TLR2 agonist).

As extended treatment of mice with Pam_3_CSK_4_ caused relevant changes in the spleen (see Figure 3C), and we have previously shown that TLR2 ligands induce myeloid differentiation of HSPCs *in vivo* (Megías et al., 2012), we next examined the number (Figure 4B, Supplementary Figure 2) and functional phenotype (Figure 4C) of mature myeloid cells in the spleen of Pam_3_CSK_4_-treated mice. Treated mice showed a marked increase in mature myeloid cells in the spleen, as both the percentage and the total number of CD11b^+^ cells were significantly increased compared with control mice. This increase in CD11b^+^ cells stems from an increase in the absolute number of neutrophils, monocytes, classical dendritic cells and macrophages. Among all the increased cell types, the population that stood out the most was a population of macrophages (CD11b^+^, MHCII^+^, Ly6C^−^ and CD11c^−^) that raised from 1.5% in control mice to 13.6% in Pam_3_CSK_4_ treated mice (Figure 4B, Supplementary Figure 2). Next, we analyzed *in vitro* the cytokine production and the fungicidal activity of total splenocytes from control and Pam_3_CSK_4_-treated mice. The production of TNF-α in response to Pam_3_CSK_4_ or LPS was significantly diminished in total splenocytes from Pam_3_CSK_4_-treated mice compared to control mice splenocytes, indicating that mature myeloid cells in the spleen of treated mice are tolerized. Splenocytes from Pam_3_CSK_4_-treated mice showed higher fungicidal activity than splenocytes from control mice. This result correlates with the higher number of mature myeloid cells (mostly phagocytic cells) in the spleen of treated mice, but may also indicate a higher fungicidal activity of these cells due to the TLR2 ligand treatment. Therefore, after an extended *in vivo* TLR2 agonist exposure, myeloid cells in the spleen are expanded, tolerized and competent to kill *C. albicans* cells *in vitro*.

### Extended TLR2 agonist treatment reduces tissue invasion during systemic *C. albicans* infection

Given the marked effect of extended TLR2 agonist treatment on the phenotype of *ex vivo* produced macrophages, as well as on the accumulation of HSPCs and mature myeloid cells in the spleen, we next addressed the possibility that this treatment could influence the *in vivo* susceptibility to invasive candidiasis. C57BL/6 mice were given 100 μg of Pam_3_CSK_4_ by intraperitoneal injection at days 0, 3 and 5, and 24 h after the final dose, mice were infected via intraperitoneal injection (30 × 10^6^ yeasts per mouse) of a virulent strain of *C. albicans* (Figure 4A). Four days post-infection, fungal burden in the kidney and spleen were determined (Figure 4D). Pam_3_CSK_4_-treated mice showed a marked decrease in CFUs in both organs, as compared with infected control mice. This result clearly indicates that an extended TLR2 agonist treatment protects mice from tissue invasion during systemic *C. albicans* infection.

### Immunodepletion of C-Kit^+^ progenitors in TLR2 agonist-treated mice abrogates protection against fungal tissue invasion during candidiasis and impacts the cytokine production by splenocytes

**Figure 5:**
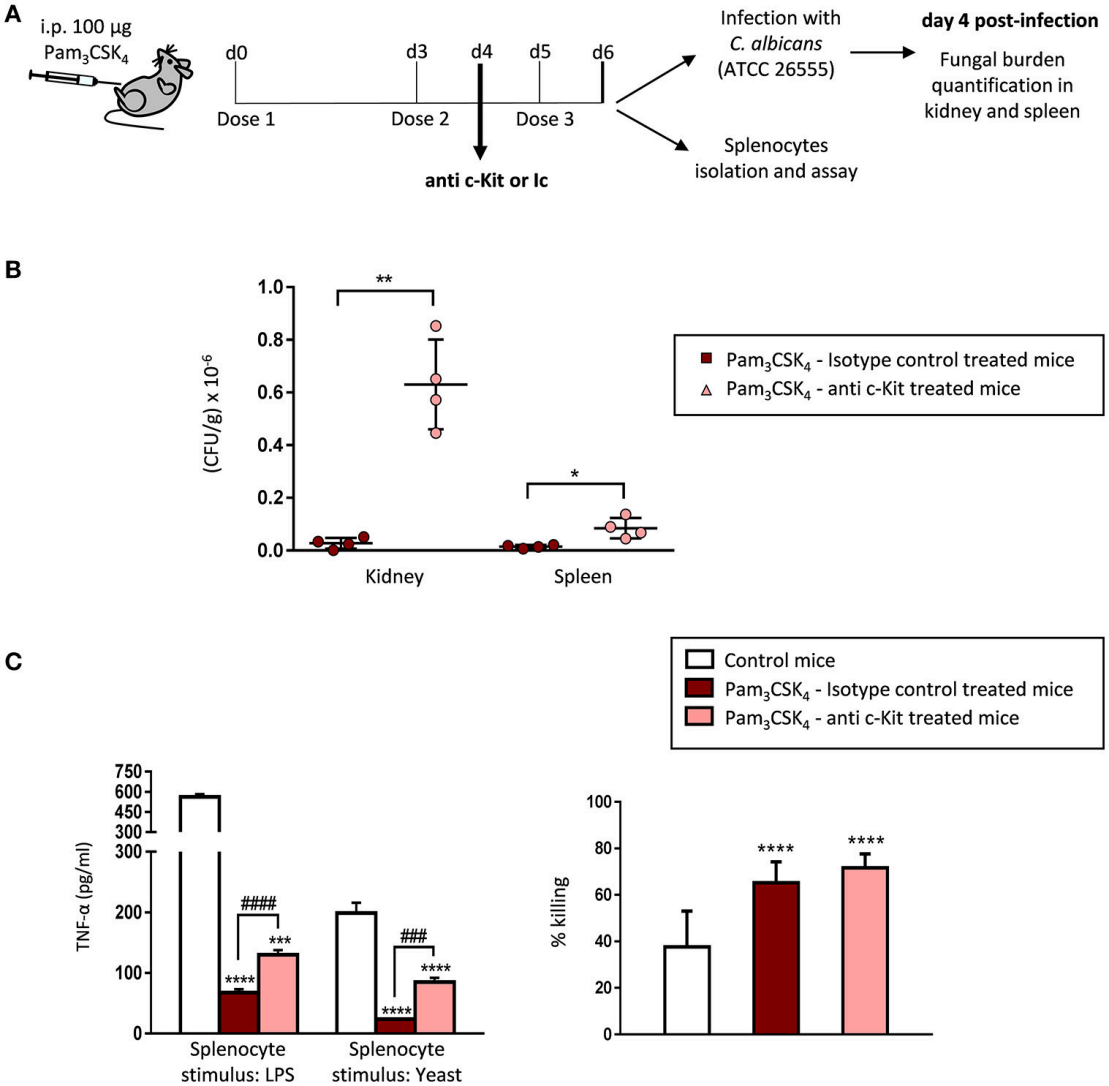
Immunodepletion of c-Kit^+^ progenitors in TLR2 agonist-treated mice abrogates protection against candidiasis and impacts the cytokine production by splenocytes. **(A)** Schematic protocol (as described in section Materials and Methods). WT mice were given 100 μg of Pam_3_CSK_4_ by intraperitoneal injection at days 0, 3, and 5 (3 doses), and 500 μg of the anti-c-Kit antibody or isotype control at day 4. 24 h after the final dose of Pam_3_CSK_4_, mice were injected intraperitoneally with 30 × 10^6^ yeasts of *C. albicans* ATCC 26555, or were sacrificed to isolate the spleen. Four days post-infection mice were sacrificed to assess the outgrowth of the yeasts in the kidney and spleen. **(B)** The fungal burden in kidneys and spleens are expressed as CFUs per gram of tissue. Results are expressed as means ± SD of pooled data from two experiments (*n* = 2 mice each group per experiment). *P < 0.05 and **P < 0.01 with respect to control mice (treated with the TLR2 agonist and isotype control). **(C)** TNF-α production and fungicidal activity of splenocytes. Splenocytes were plated at a density of 2.5 × 10^6^ cells in 0.5 ml of complete cell culture medium. For cytokine assays, splenocytes were challenged with LPS (100 ng/ml) or inactivated yeasts of *C. albicans* (2 × 10^6^ yeasts/ml) for 24 h. TNF-α levels in cell-free culture supernatants were measured by ELISA. For fungicidal activity determination, splenocytes were challenged with 100,000 viable PCA2 yeasts and incubated for 4 h. After incubation, samples were diluted, plated on Sabouraud dextrose agar and incubated overnight at 37°C; CFUs were counted and killing percentages were determined as follows: % killing = [1 – (CFUs sample at *t* = 4 h)/(CFUs control at *t* = 4 h)] × 100. Triplicate samples were analyzed in each assay. Results are expressed as means ± SD of pooled data from two experiments. ***P < 0.001 and ****P < 0.0001 with respect to control cells (splenocytes from control mice), and ^###^*P* < 0.001 and ^####^*P* < 0.0001 with respect to cytokine production by splenocytes from mice treated with the TLR2 agonist and isotype control.

To determine whether HSPCs are required for TLR2 agonist mediated protection against tissue invasion, we immunodepleted c-Kit^+^ progenitors in Pam_3_CSK_4_-treated mice before *C. albicans* infection. For this purpose, we used the monoclonal antibody ACK2, as it has previously been reported that the complete inhibition of c-Kit signaling by ACK2 administration *in vivo* leads to the rapid but transient depletion of bone marrow HSCs (95% depletion at 48 h post-injection, with a maximum of depletion at day 9, and a recovery to normal levels at day 23) (Czechowicz et al., 2007). Taking into account these data, in our model of extended TLR2 agonist treatment, mice were given 500 μg of ACK2 or its isotype control, by intraperitoneal injection, at day 4 (48 h before infection) (Figure 5A). As shown in Supplementary Figure 3, the injection of ACK2 at day 4 results in a significant reduction of Lin^−^ c-Kit^+^ progenitors at day 6, both in the bone marrow (roughly 56% reduction) and in the spleen (roughly 75% reduction) as compared with isotype control injected animals. Despite of HSPCs depletion, similar amounts of mature myeloid cells were found in the spleen of immunodepleted mice compared to isotype control injected mice (data not shown).

We then assessed the fungal burden in the kidney and spleen 4 days post infection (Figure 5B). ACK2-treated mice showed a marked increase in CFUs in both organs, as compared with isotype control injected mice, clearly demonstrating that depletion of c-Kit^+^ progenitors in Pam_3_CSK_4_-treated mice abrogates protection against tissue invasion during invasive candidiasis. Therefore, we conclude that the TLR2 agonist induced protection against candidiasis is at least partially mediated by the expansion of hematopoietic progenitors.

*In vitro* cytokine production and fungicidal activity of total splenocytes from control and c-Kit depleted mice were also analyzed (Figure 5C). As expected, mature myeloid cells in the spleen of Pam_3_CSK_4_-treated mice were tolerized, as the production of TNF-α in response to LPS or inactivated yeasts was significantly diminished in total splenocytes from Pam_3_CSK_4_-treated mice compared to splenocytes from control (untreated) mice. Interestingly, splenocytes from c-Kit depleted animals produced significantly higher amounts of TNF-α than the isotype control treated mice splenocytes, although still lower levels than splenocytes from control (untreated) mice. The increased splenocyte fungicidal activity induced by the Pam_3_CSK_4_ treatment was similar in c-Kit depleted mice and isotype control injected mice. In conclusion, depletion of c-Kit^+^ progenitors in Pam_3_CSK_4_-treated mice increases the cytokine production by splenocytes without modifying their potentiated fungicidal activity.

### HSPCs produce cytokines and chemokines in response to *C. albicans* and Pam_3_CSK_4_

**Figure 6:**
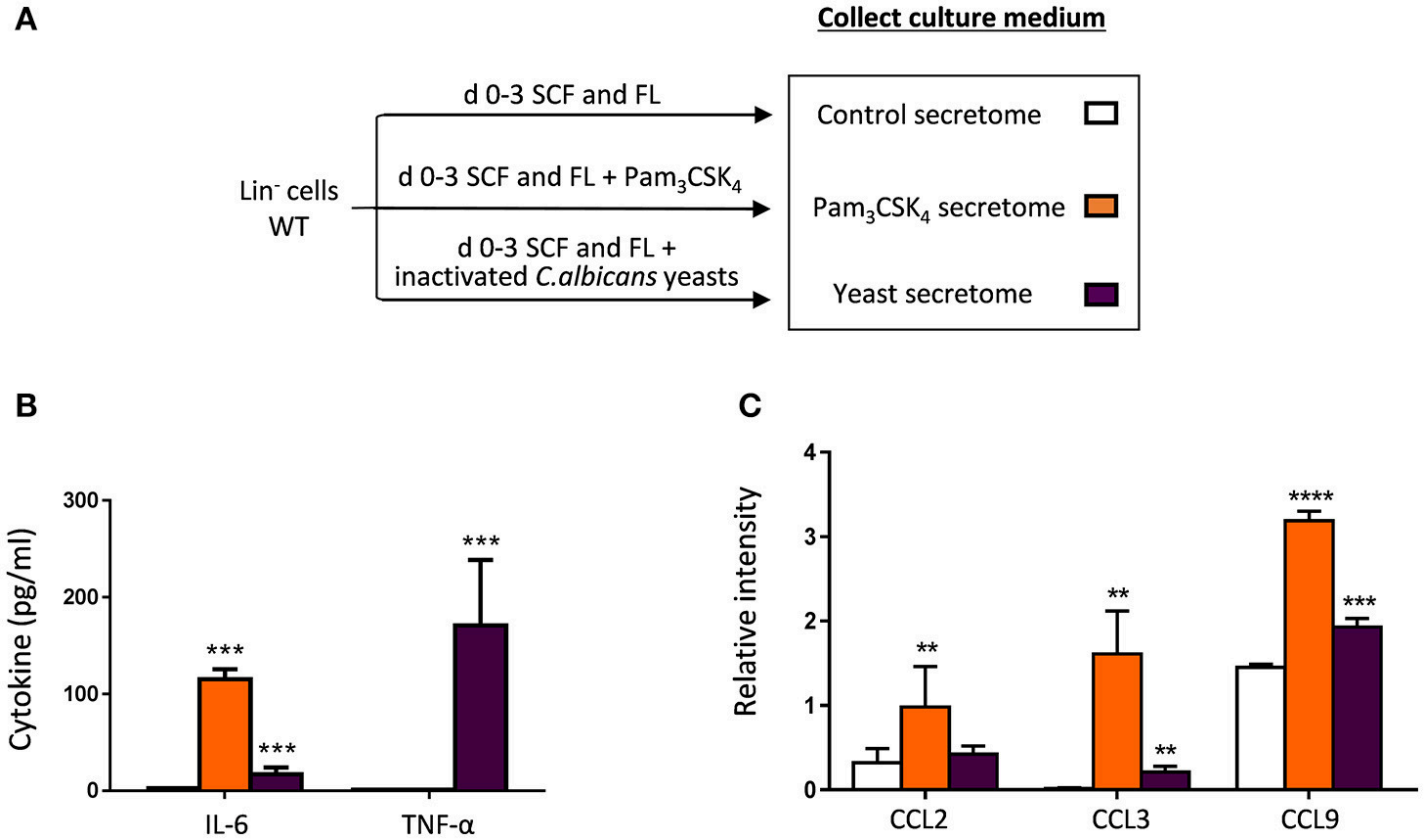
Cytokine production by HSPCs in response to *C. albicans* and the TLR2 agonist. **(A)** Schematic protocol (as described in section Materials and Methods) to obtain secretomes. WT Lin^−^ cells were cultured at a density of 50,000 cells in 250 μl of a serum-free medium containing FL and SCF, in the presence or absence of Pam_3_CSK_4_ or inactivated *C. albicans* yeasts for 3 days. Then, secretomes (conditioned media) were collected. **(B)** TNF-α and IL-6 levels in the secretomes were measured by ELISA. **(C)** CCL2, CCL3, and CCL9 levels were determined using a mouse cytokine array (as described in section Materials and Methods). Data are expressed as means ± SD of pooled data from two experiments. **P < 0.01, ***P < 0.001, and ****P < 0.0001 with respect to control secretome.

The above described findings indicate that HSPCs sense the TLR2 agonist and *C. albicans* yeasts *in vivo*, and subsequently may contribute to protect the host against candidiasis. These results prompted us to investigate potential mechanisms through which HSPCs could protect mice against infection. Since it has been described that HSPCs produce cytokines in response to LPS and Pam_3_CSK_4_ (Zhao et al., 2014), we profiled the molecules secreted by HSPCs in response to *C. albicans* yeasts and Pam_3_CSK_4_. Lin^−^ cells were cultured in a serum-free medium, in the absence (control) or presence of inactivated yeasts or Pam_3_CSK_4_, and 3 days later the culture supernatants (secretomes) were collected and analyzed (Figure 6A). First, TNF-α and IL-6 levels were measured in the secretomes using quantitative ELISAs (Figure 6B). The levels of cytokines in control secretome were undetectable. Consistent with a previous report (Zhao et al., 2014) HSPCs produced IL-6 in response to the TLR2 ligand. IL-6 and high amounts of TNF-α were produced in response to inactivated *C. albicans* yeasts. Next, 40 cytokines were determined in the secretomes using a mouse cytokine detection array (Figure 6C, Supplementary Figure 4). Representative cytokine array blots are shown in Supplementary Figure 4. Figure 6C show values of the relative intensity compared to array positive controls; only those cytokines whose values were significantly different among secretomes are depicted. The results showed that HSPCs produce three chemokines, CCL2, CCL3, and CCL9, in response to the TLR2 ligand, while only CCL3 and CCL9 were higher in the yeast secretome, compared to control secretome. Overall, these data demonstrate that HSPCs are capable of producing proinflammatory cytokines and chemokines in response to different PRR ligands.

### HSPC secretomes in response to *C. albicans* and TLR2 agonist induce myeloid differentiation and modulate macrophage cytokine production

**Figure 7:**
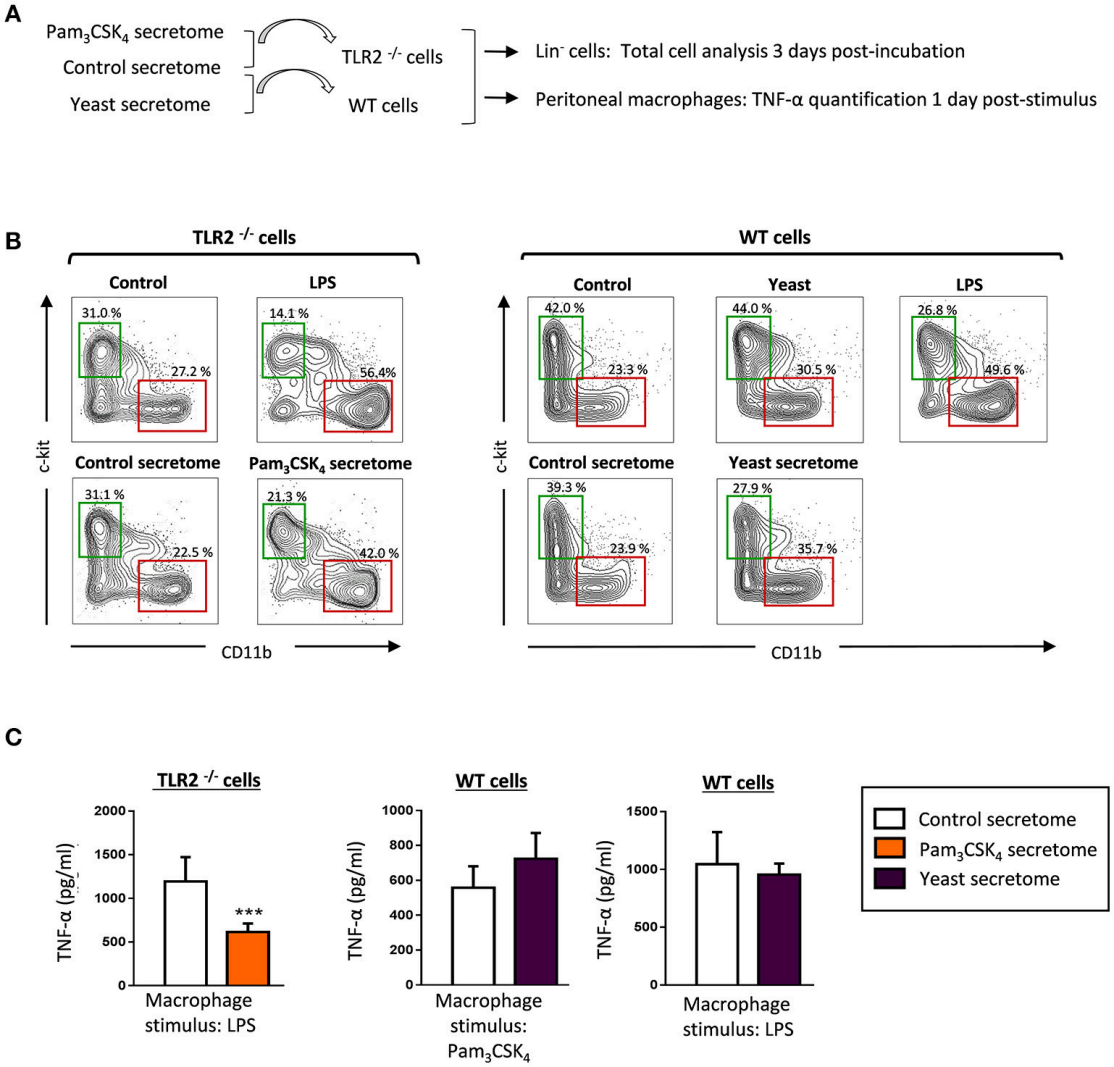
HSPC secretomes in response to *C. albicans* and the TLR2 agonist induce myeloid differentiation and modulate cytokine production by macrophages. **(A)** Schematic protocol (as described in section Materials and Methods) to study the effect of secretomes (obtained as indicated in Figure 5A) on HSPC differentiation and the production of TNF-α by peritoneal macrophages. HSPCs or peritoneal macrophages from TLR2 ^−/−^ mice were used to study the effect of the secretomes from HSPCs stimulated with Pam_3_CSK_4_. **(B)** Lin^−^ cells were cultured at a density of 50,000 cells in 250 μl of secretomes for 3 days, labeled with antibodies, and analyzed by flow cytometry. Cells were gated as c-Kit or CD11b positive cells. The indicated percentages refer to total analyzed cells. Results shown are from one representative of two independent experiments. **(C)** Resident peritoneal macrophages were cultured at a density of 150,000 cells in 250 μl of the indicated secretomes, and challenged with Pam_3_CSK_4_ (100 ng/ml) or LPS (100 ng/ml) for 24 h. TNF-α levels in cell-free culture supernatants were measured by ELISA. Triplicate samples were analyzed in each assay. Results are expressed as means ± SD of pooled data from two experiments. ***P < 0.001 with respect to cytokine production by peritoneal macrophages in the presence of control secretome.

The above data demonstrate that HSPCs produce and secrete diverse cytokines in response to a TLR2 agonist and *C. albicans* yeasts. Hence, we wondered whether the secretomes may play some role in regulating the function of surrounding cells, such as HSPCs or resident macrophages. First, we examined the effect of secretomes on HSPC differentiation (Figures 7A,B). To study the effects of secretomes from Pam_3_CSK_4_- stimulated HSPCs, we used cells from TLR2^−/−^ mice to avoid stimulation by soluble Pam_3_CSK_4_ present in the secretomes. Lin^−^ cells were cultured in the presence of secretomes for 3 days, and expression of CD11b (a marker of myeloid differentiation) and c-Kit (a marker of progenitor cells) was analyzed by flow cytometry. Lin^−^ cells were also cultured with LPS or inactivated yeasts, as positive controls of differentiation. The differentiation pattern in the presence of control secretomes was similar to the control samples (cells cultured in fresh medium). However, Lin^−^ cells cultured in the presence of Pam_3_CSK_4_ secretomes or yeast secretomes increased the percentage of CD11b^+^ cells, as compared to the control secretomes (42 vs. 22.5%, for the Pam_3_CSK_4_ secretomes, and 35.7 vs. 23.9% for the yeast secretomes). Accordingly, the percentage of c-Kit^+^ cells decreased as compared to the control secretomes (21.3 vs. 31.1% for the Pam_3_CSK_4_ secretomes, and 27.9 vs. 39.3% for the yeast secretomes). Therefore, HSPC secretomes in response to *C. albicans* and the TLR2 agonist induce myeloid differentiation of Lin^−^ progenitors.

Finally, we studied the effect of secretomes on the cytokine production by tissue resident macrophages (Figures 7A,C). Resident peritoneal macrophages from TLR2^−/−^ mice or wild-type C57BL/6 mice were used to test the Pam_3_CSK_4_ secretome or the yeast secretome, respectively. TNF-α production in response to LPS was significantly decreased in the presence of the Pam_3_CSK_4_ secretome, whereas the yeast secretome had no effect on the cytokine production by macrophages in response to Pam_3_CSK_4_ or LPS.

Taken together, these results show that detection of PAMPs by HSPCs defines the secretome they produce, and therefore its impact on HSPC differentiation and macrophage function. These results support the idea that direct pathogen sensing by HSPCs plays an active role in regulating the immune responses against infection.

## Discussion

We have previously reported that PRR-mediated recognition of *C. albicans* by HSPCs, both *in vitro* and *in vivo*, induces myeloid differentiation, and therefore may help replace or increase cells that constitute the first line of defense against fungus (Yáñez et al., 2009, 2010, 2011; Megías et al., 2013). The direct interaction of *C. albicans* with HSPCs may involve yeast cells and/or fungal derived PAMPs in the bone marrow. In a previous report we demonstrated the presence of viable yeasts in the bone marrow of mice infected with a low virulence strain (Yáñez et al., 2011), and it is well known that some fungal PAMPs such as mannan and glucan are present in the blood of patients with systemic candidiasis (López-Ribot et al., 2004; Ostrosky-Zeichner et al., 2005). In addition this interaction may also occur outside the bone marrow, as HSPCs may be mobilized in response to inflammation and or infection (Yáñez et al., 2013a). However, to elucidate whether this new mechanism by which HSPCs sense pathogens may be protective, the study of the functional properties of the generated myeloid cells is essential. In this context, our previous *in vitro* studies have shown that detection of PAMPs by HSPCs impacts the antimicrobial function of the macrophages they produce (Megías et al., 2016; Martínez et al., 2017). HSPC activation in response to *C. albicans* leads to the generation of inflammatory macrophages better prepared to deal with the infection: they produce higher levels of cytokines and are better at killing yeasts than M-CSF-derived macrophages produced in homeostatic conditions. These *in vitro* results suggest that the concept of “trained innate immunity” may apply not only to differentiated cells but also to HSPCs (Megías et al., 2016).

In this work, we used an *ex vivo* model to further investigate whether HSPCs may sense *C. albicans* during infection *in vivo*, and whether this impacts the antimicrobial function of the macrophages they produce *in vitro*. We have shown here that early during candidiasis, the *ex vivo* cultured HSPCs give rise to macrophages with a trained phenotype in their cytokine response to a TLR2 ligand and with a higher fungicidal activity. However, when the infection progresses to high fungal burden levels, the *ex vivo* HSPCs-derived macrophages become tolerized in their cytokine response, while they keep up their fungicidal capacity. We can deduce from the phenotype of the generated macrophages that this could be beneficial for the host during infection. In the first stages of the infection, inflammatory cytokines and phagocytes with high fungicidal capacity are needed, whereas when the fungal invasion progresses, a “cytokine storm” could be harmful, and therefore the macrophages generated are tolerized. The reasons for the opposite responses (training or tolerance) during infection are not obvious. However, it can be speculated that it could be related to the increasing pathogen loads during infection, to the progressive tissue damage and/or impairment of immune cell function, which shift from fighting the pathogen (training) toward maintenance and repair activities leading probably toward a phenotype of tolerance to pathogen. The fact that pathogen dose plays a key role in determining hormetic responses has been recently proposed for mature innate immune cells (Bauer et al., 2018). Our results suggest that this hormetic response also occurs in HSPCs during infection. The tolerized response would only develop when the early trained response is not able to deal with the infection and, therefore, pathogen burden increases. Although the *in vitro* generated phenotype in our *ex vivo* model may not exactly reproduce the *in vivo* situation, our results clearly indicate that HSPCs sense the infection *in vivo*, which profoundly alters the functional phenotype of the macrophages derived from them *ex vivo*.

In the opposite direction to trained immunity, Pam_3_CSK_4_ tolerized phenotype in macrophages is a well-known mechanism that avoids sustained activation, which can be detrimental to the host (Medvedev et al., 2006). Our previous studies have shown that macrophages produced by HSPCs exposed to Pam_3_CSK_4_, prior to or during macrophage differentiation, exhibit a reduced inflammatory cytokine response, demonstrating that Pam_3_CSK_4_-induced tolerance also occurs in HSPCs (Yáñez et al., 2013b; Megías et al., 2016; Martínez et al., 2017). Moreover, fungal ligands partially reversed the Pam_3_CSK_4_ tolerized phenotype of M-CSF-derived macrophages *in vitro* (Martínez et al., 2017). Now, we show that a short systemic TLR2 agonist exposure *in vivo* results in tolerized HSPCs-derived macrophages *ex vivo* and that the development of this tolerized phenotype is partially reversed by *C. albicans* yeasts during the *in vitro* differentiation culture. Therefore, this *ex vivo* model is consistent with our previously reported *in vitro* results (Megías et al., 2016; Martínez et al., 2017). However, an extended systemic TLR2 agonist exposure *in vivo* generates HSPC-derived macrophages that produce higher amounts of cytokines (trained macrophages) than control macrophages. This extended TLR2 agonist exposure leads to an expansion of spleen HSPCs, as previously described by Herman et al. (2016). These spleen HSPCs, similarly to bone marrow HSPCs, also generate *in vitro* trained macrophages. Therefore, the *ex vivo* tolerized or trained phenotype depends on the dose and timing of the signals (direct TLR2-mediated signaling and cytokines released by different cell types in response to Pam_3_CSK_4_) that HSPCs receive *in vivo*. It should be noted that the TLR2 agonist exposure and *C. albicans* infection are not comparable models. Therefore, the observed opposite responses of HSPCs to Pam_3_CSK_4_, switching from tolerance (one dose) to training (extended exposure), cannot be related to pathogen load, as the response to Pam_3_CSK_4_ involves only one PRR (TLR2) and occurs in the absence of deleterious effects associated to infection. Interestingly, mature myeloid cells are tolerized in the spleen of these animals, probably due to the direct effect of the *in vivo* administrated TLR2 ligand on mature macrophages. In addition, the fungicidal activity of the *ex vivo* generated macrophages was also dependent on the dose and timing of the signals that HSPCs receive *in vivo*, as well as on the source of the HSPCs (bone marrow or spleen). Even though there is not a clear relationship between tolerized or trained macrophages and their killing activity.

These results demonstrate that the memory-like innate immune training or tolerance, already described for monocytes and macrophages, also occurs in HSPCs, and suggest that the tailored manipulation of these responses may serve as an immunotherapeutic approach to boost the innate immune response to infection. These memory-like innate immune responses probably also occur during infections other than candidiasis, and development of training or tolerance may depend on the severity and time-course of infection (microbial burden and associated deleterious effects), as well as on the PAMPs expressed by specific pathogens.

The relationship between the *ex vivo* phenotype of the generated macrophages and their role during infection is not obvious. Therefore, it is difficult to predict the influence of extended Pam_3_CSK_4_ treatment on the susceptibility to infection. We hypothesized that the substantial accumulation of HSPCs and mature myeloid cells in the spleen could protect mice against candidiasis, and that HSPCs may contribute to this protection, probably by providing an additional site for the generation of effector cells during infection. Results clearly indicate that the extended TLR2 agonist treatment strongly protects mice from tissue invasion during systemic *C. albicans* infection, as the fungal burden in the kidney and in the spleen was drastically reduced. Myeloid cells expand in the spleen of Pam_3_CSK_4_ treated mice, being macrophages the most amplified population; therefore, it would be possible to suggest that these myeloid cells could be responsible for protection. However, immunodepletion of c-Kit^+^ progenitors in TLR2 agonist-treated mice abrogates protection against tissue invasion during candidiasis, despite their similar amount of mature myeloid cells in the spleen at the moment of infection, than isotype control injected mice. In conclusion, the protective effect against candidiasis in Pam_3_CSK_4_-treated mice is at least partially dependent on HSPCs. These results are in line with Granick et al. (2013), which reported that proliferation of HSPCs in skin wounds in response to *Staphylococcus aureus* is TLR2-mediated and contributes significantly to the production of neutrophils and resolution of local infection, supporting a role for TLR2 signaling in the regulation of extramedullary hematopoiesis. Other authors have also described that systemic infection of mice with *Escherichia coli* causes mobilization of functional HSPCs to the spleen, and that mobilized HSPCs give rise to neutrophils and monocytes and contribute to limiting secondary infection (Burberry et al., 2014). The possibility that protection against candidiasis in our model of Pam_3_CSK_4_-treated mice may be also mediated by mature myeloid cells cannot be completely excluded. Supporting this possibility, Wang et al. (2003) have shown that tolerance induced by one dose of a TLR2 ligand protects mice against live *S. aureus* and *Salmonella typhimurium* and they suggested that this protection is mediated by an increased bacterial recognition and bactericidal activity of neutrophils and macrophages in these mice.

A growing body of evidence has emerged supporting a role for HSPCs in the fight against infection, yet mechanisms governing the response of HSPCs to infection are poorly understood (Yáñez et al., 2013a; Kobayashi et al., 2016; Boettcher and Manz, 2017). Various mechanisms are involved in HSPCs responses to infection, including cytokine signaling, egressing to peripheral tissues, and direct sensing of PAMPs by HSPCs themselves (Kobayashi et al., 2016). The effector mechanisms of HSPCs for protection against candidiasis in the Pam_3_CSK_4_-treated mice are not well defined yet, although our data point toward the involvement of diverse mechanisms. In agreement with Zhao et al. (2014), we confirm here that HSPCs are capable of directly responding to TLR ligands by producing cytokines to coordinate immune responses. Moreover, we show for the first time that HSPCs also produce the CCL2, CCL3 and CCL9 chemokines in response to different PRRs ligands. Chemokines involved in the early recruitment of innate immune cells to the sites of infection are crucial for local control of fungal infections (Lionakis and Levitz, 2017). Consequently, CCL2, CCL3, and CCL9 produced by HSPCs may activate and attract neutrophils, monocytes and dendritic cells, therefore contributing to another level of protection against infection. Moreover, it has been described that HSPCs express CCR2 and that this receptor mediates recruitment of these HSPCs to sites of inflammation (Si et al., 2010), therefore it could be speculated that HSPCs may induce their own recruitment. In addition, we show that HSPCs secretomes produced in response to the TLR2 ligand and *C. albicans* yeasts induce Lin^−^ myeloid differentiation. Zhao et al. (2014) described that among the cytokines produced by HSPCs in response to LPS and Pam_3_CSK_4_, IL-6 is a particularly important regulator of myeloid differentiation. Our results suggest that other secreted molecules by HSPCs in response to *C. albicans* may induce myeloid differentiation, as IL-6 was barely found in the yeast secretome. Secretomes acting in a paracrine or autocrine manner on HSPCs may mediate a rapid myeloid cell recovery during candidiasis. Finally, in addition to acting on HSPCs, secretomes may also modulate the function of other surrounding cells, such as macrophages. In this context, we found that the Pam_3_CSK_4_ secretome reduces the TNF-α production by peritoneal macrophages. In agreement with this *in vitro* effect, the production of cytokines by splenocytes from c-Kit^+^ depleted mice is increased as compared to isotype control treated mice. Therefore, the Pam_3_CSK_4_-mobilized HSPCs may contribute to protection against infection, not only by myeloid cell replenishment but also by secreting molecules that recruit and activate leukocytes, and modulate the phenotype of mature myeloid cells. Future studies will be required to identify the molecules and the mechanisms responsible for the different secretome functions.

In conclusion, our data show that HSPCs sense candidiasis and this profoundly alters the functional phenotype of the macrophages *ex vivo* derived from them. Extended systemic exposure to TLR2 agonist leads to an expansion of spleen HSPCs that are, at least partially, responsible for protection against tissue invasion during systemic *C. albicans* infection. HSPCs produce cytokines and chemokines in response to *C. albicans* and TLR2 agonist, and these secreted molecules induce myeloid differentiation of HSPCs and modulate cytokine production by peritoneal macrophages. Our results support the hypothesis that HSPCs can sense pathogens during infection and contribute to protect the host by several mechanisms. A better understanding of the signals that influence HSPCs during infection may lead to new therapeutic strategies for anti-infection intervention.

## Author contributions

MG and DG designed the study. AM, CB, and JM performed the experiments. AM, JM, AY, MG, and DG analyzed and interpreted data. MG and DG wrote the manuscript. All the authors read, revised, and approved the final version of the manuscript.

### Conflict of interest statement

The authors declare that the research was conducted in the absence of any commercial or financial relationships that could be construed as a potential conflict of interest.
