# Supplementary material for: Systemic Candidiasis and TLR2 Agonist Exposure Impact the Antifungal Response of Hematopoietic Stem and Progenitor Cells
Source: Front Cell Infect Microbiol. 2018 Sep 3;8:309. doi: 10.3389/fcimb.2018.00309 (PMC6130230; doi:10.3389/fcimb.2018.00309)
Supplement: Supplementary file 1 [file Presentation_1.PPTX]

## Slide 1
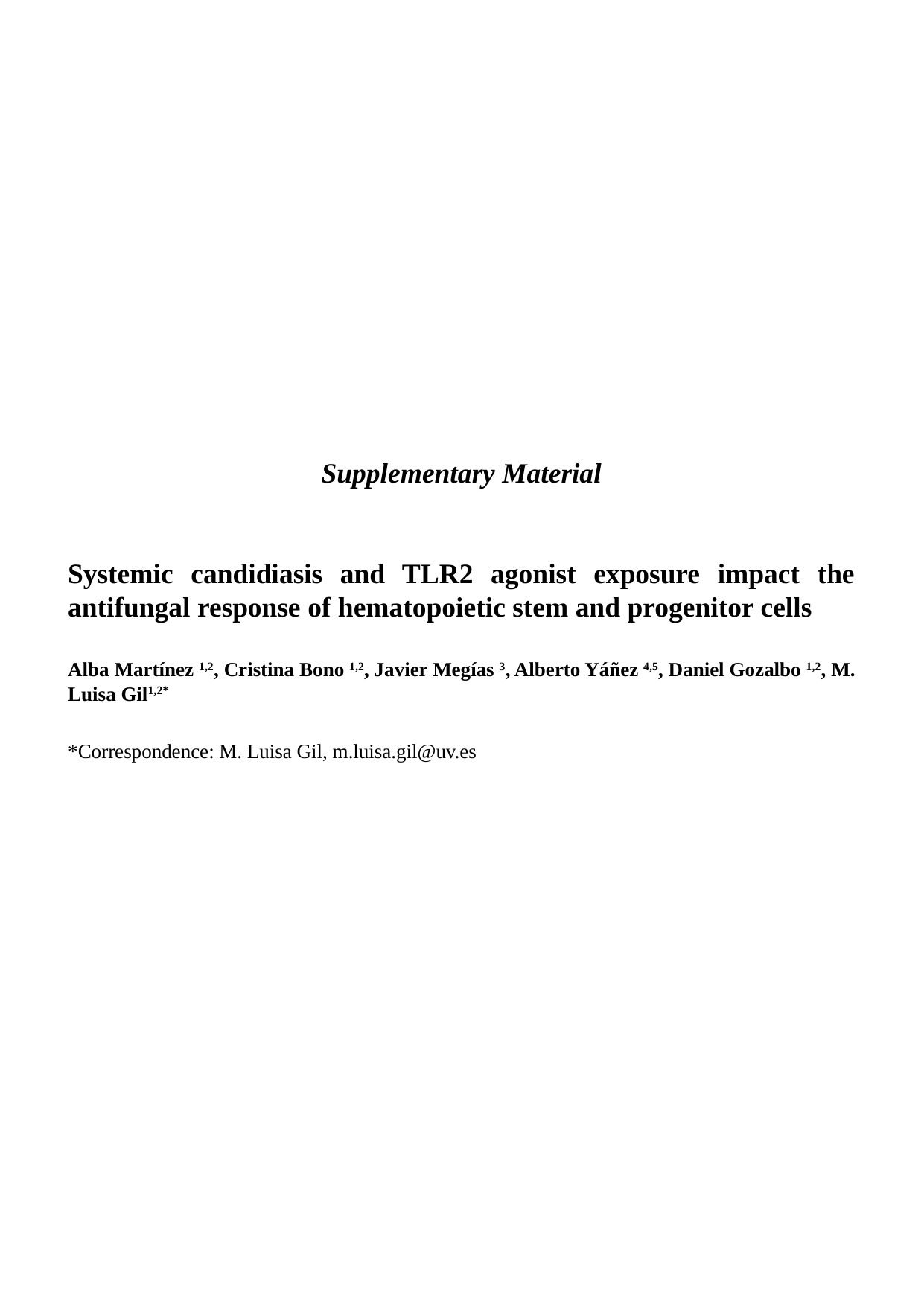

Supplementary Material
Systemic candidiasis and TLR2 agonist exposure impact the antifungal response of hematopoietic stem and progenitor cells
Alba Martínez 1,2, Cristina Bono 1,2, Javier Megías 3, Alberto Yáñez 4,5, Daniel Gozalbo 1,2, M. Luisa Gil1,2*
*Correspondence: M. Luisa Gil, m.luisa.gil@uv.es

## Slide 2
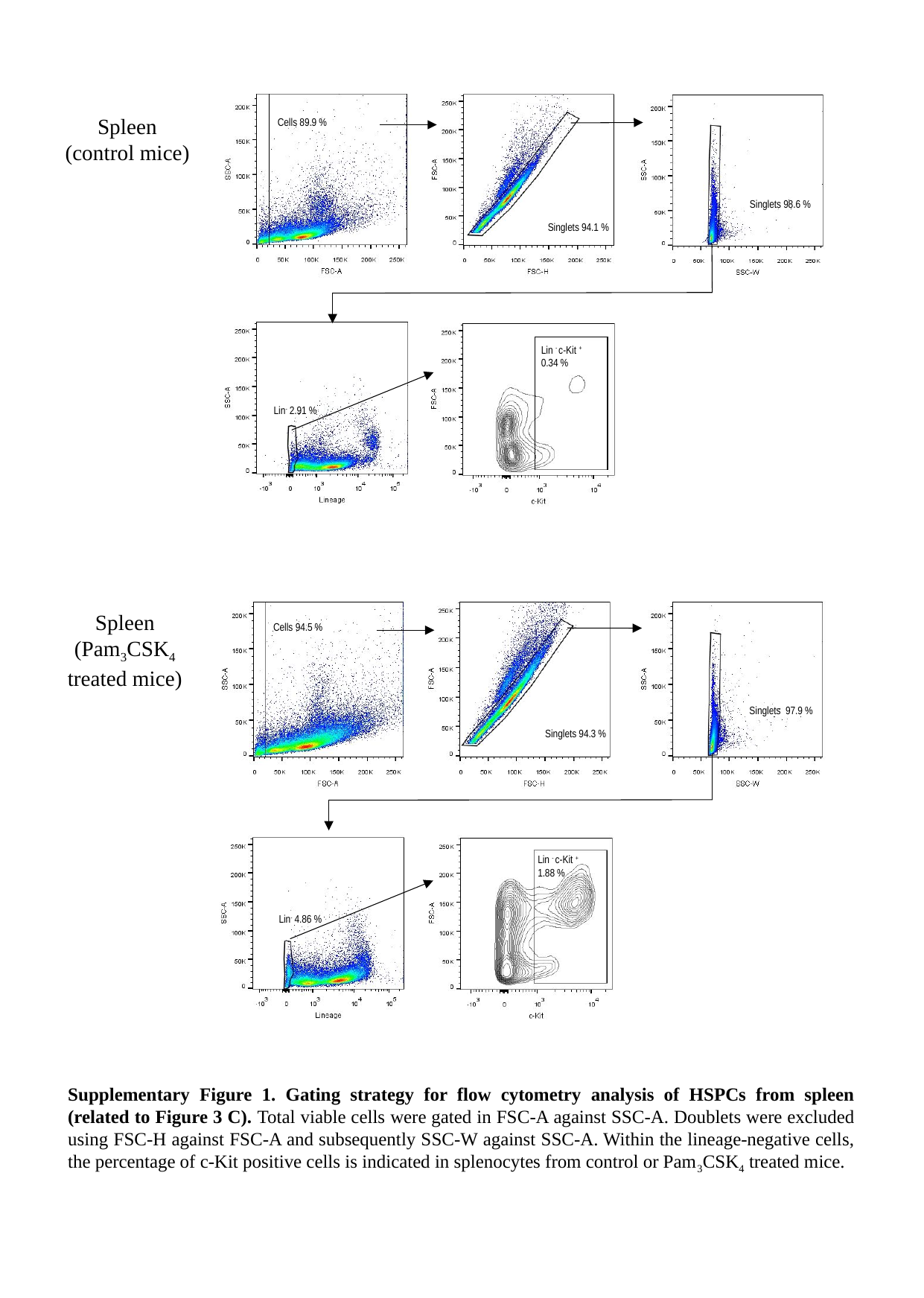

Cells 89.9 %
Singlets 98.6 %
Singlets 94.1 %
Lin - c-Kit + 0.34 %
Lin- 2.91 %
Spleen (control mice)
Cells 94.5 %
Singlets 97.9 %
Singlets 94.3 %
Lin - c-Kit + 1.88 %
Lin- 4.86 %
Spleen (Pam3CSK4 treated mice)
Supplementary Figure 1. Gating strategy for flow cytometry analysis of HSPCs from spleen (related to Figure 3 C). Total viable cells were gated in FSC-A against SSC-A. Doublets were excluded using FSC-H against FSC-A and subsequently SSC-W against SSC-A. Within the lineage-negative cells, the percentage of c-Kit positive cells is indicated in splenocytes from control or Pam3CSK4 treated mice.

## Slide 3
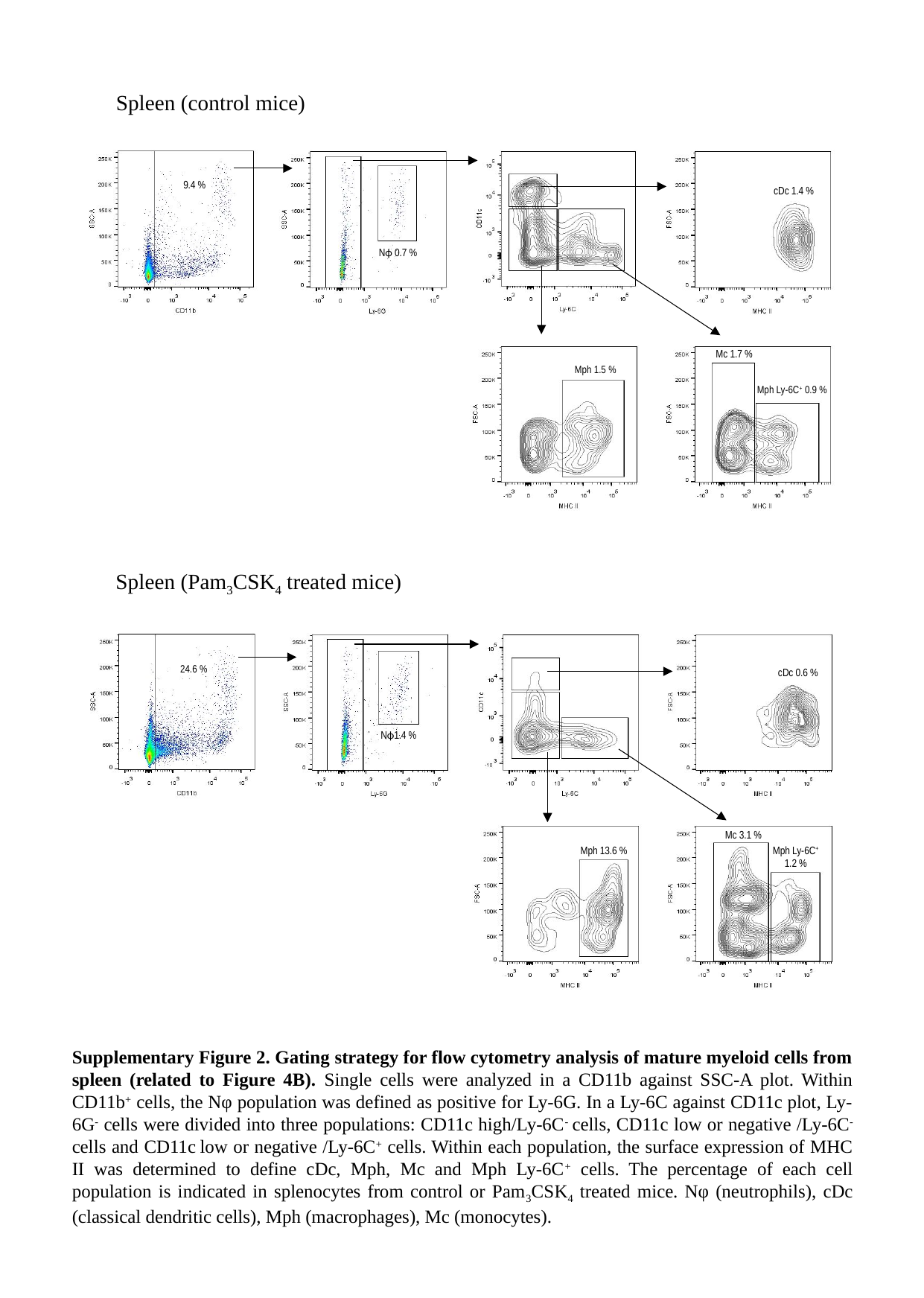

Spleen (control mice)
9.4 %
cDc 1.4 %
 Nϕ 0.7 %
Mc 1.7 %
Mph 1.5 %
Mph Ly-6C+ 0.9 %
Spleen (Pam3CSK4 treated mice)
24.6 %
cDc 0.6 %
 Nϕ1.4 %
Mc 3.1 %
Mph 13.6 %
Mph Ly-6C+ 1.2 %
Supplementary Figure 2. Gating strategy for flow cytometry analysis of mature myeloid cells from spleen (related to Figure 4B). Single cells were analyzed in a CD11b against SSC-A plot. Within CD11b+ cells, the Nφ population was defined as positive for Ly-6G. In a Ly-6C against CD11c plot, Ly-6G- cells were divided into three populations: CD11c high/Ly-6C- cells, CD11c low or negative /Ly-6C- cells and CD11c low or negative /Ly-6C+ cells. Within each population, the surface expression of MHC II was determined to define cDc, Mph, Mc and Mph Ly-6C+ cells. The percentage of each cell population is indicated in splenocytes from control or Pam3CSK4 treated mice. Nφ (neutrophils), cDc (classical dendritic cells), Mph (macrophages), Mc (monocytes).

## Slide 4
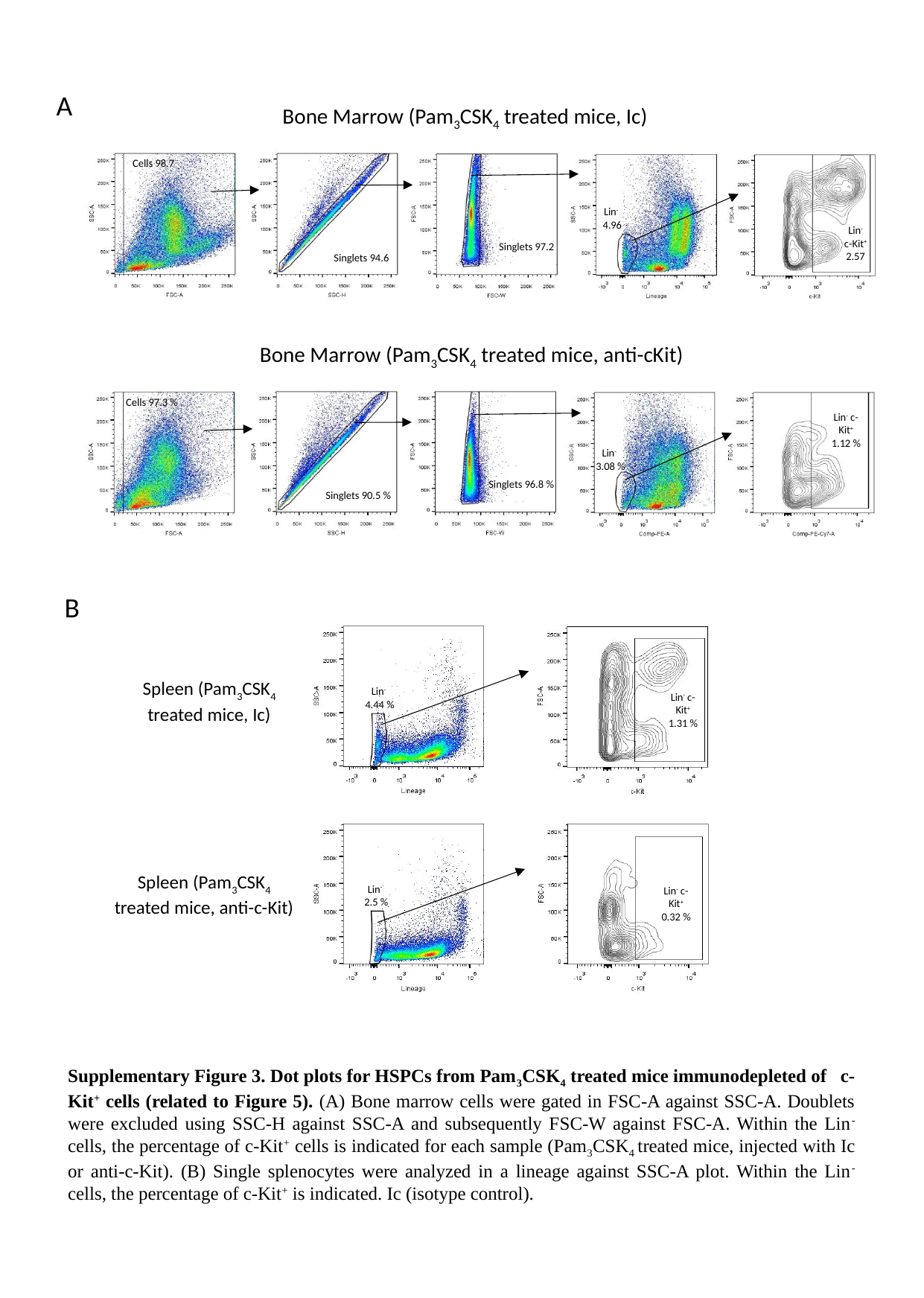

A
Bone Marrow (Pam3CSK4 treated mice, Ic)
Cells 98.7
Lin- 4.96
Lin- c-Kit+ 2.57
Singlets 97.2
Singlets 94.6
Bone Marrow (Pam3CSK4 treated mice, anti-cKit)
Cells 97.3 %
Lin- c-Kit+ 1.12 %
Lin- 3.08 %
Singlets 96.8 %
Singlets 90.5 %
B
Spleen (Pam3CSK4 treated mice, Ic)
Lin- 4.44 %
Lin- c-Kit+ 1.31 %
Spleen (Pam3CSK4 treated mice, anti-c-Kit)
Lin- 2.5 %
Lin- c-Kit+ 0.32 %
Supplementary Figure 3. Dot plots for HSPCs from Pam3CSK4 treated mice immunodepleted of c-Kit+ cells (related to Figure 5). (A) Bone marrow cells were gated in FSC-A against SSC-A. Doublets were excluded using SSC-H against SSC-A and subsequently FSC-W against FSC-A. Within the Lin- cells, the percentage of c-Kit+ cells is indicated for each sample (Pam3CSK4 treated mice, injected with Ic or anti-c-Kit). (B) Single splenocytes were analyzed in a lineage against SSC-A plot. Within the Lin- cells, the percentage of c-Kit+ is indicated. Ic (isotype control).

## Slide 5
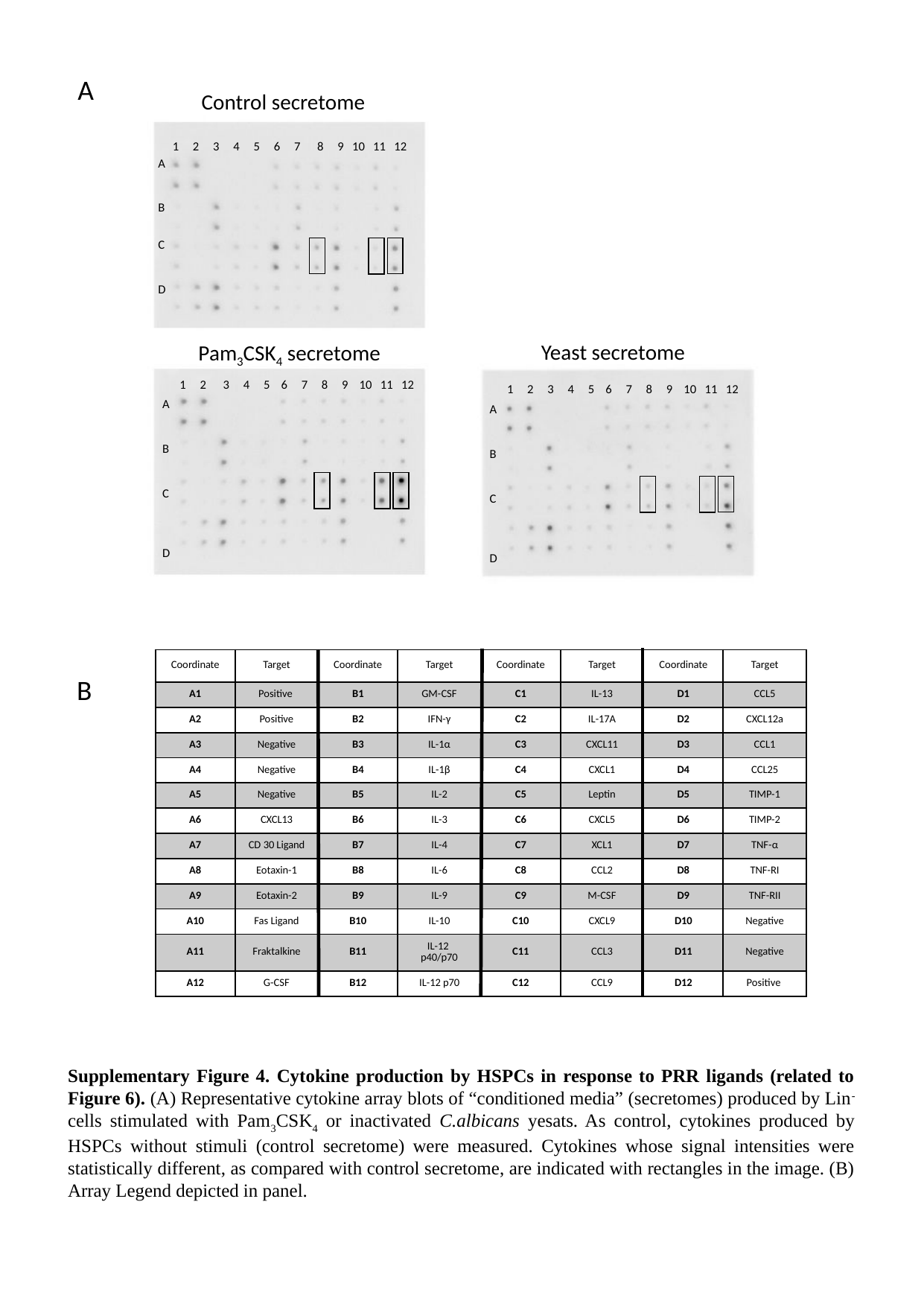

A
Control secretome
 1 2 3 4 5 6 7 8 9 10 11 12
A
B
C
D
 1 2 3 4 5 6 7 8 9 10 11 12
 1 2 3 4 5 6 7 8 9 10 11 12
A
B
C
D
A
B
C
D
Yeast secretome
Pam3CSK4 secretome
| Coordinate | Target | Coordinate | Target | Coordinate | Target | Coordinate | Target |
| --- | --- | --- | --- | --- | --- | --- | --- |
| A1 | Positive | B1 | GM-CSF | C1 | IL-13 | D1 | CCL5 |
| A2 | Positive | B2 | IFN-γ | C2 | IL-17A | D2 | CXCL12a |
| A3 | Negative | B3 | IL-1α | C3 | CXCL11 | D3 | CCL1 |
| A4 | Negative | B4 | IL-1β | C4 | CXCL1 | D4 | CCL25 |
| A5 | Negative | B5 | IL-2 | C5 | Leptin | D5 | TIMP-1 |
| A6 | CXCL13 | B6 | IL-3 | C6 | CXCL5 | D6 | TIMP-2 |
| A7 | CD 30 Ligand | B7 | IL-4 | C7 | XCL1 | D7 | TNF-α |
| A8 | Eotaxin-1 | B8 | IL-6 | C8 | CCL2 | D8 | TNF-RI |
| A9 | Eotaxin-2 | B9 | IL-9 | C9 | M-CSF | D9 | TNF-RII |
| A10 | Fas Ligand | B10 | IL-10 | C10 | CXCL9 | D10 | Negative |
| A11 | Fraktalkine | B11 | IL-12 p40/p70 | C11 | CCL3 | D11 | Negative |
| A12 | G-CSF | B12 | IL-12 p70 | C12 | CCL9 | D12 | Positive |
B
Supplementary Figure 4. Cytokine production by HSPCs in response to PRR ligands (related to Figure 6). (A) Representative cytokine array blots of “conditioned media” (secretomes) produced by Lin- cells stimulated with Pam3CSK4 or inactivated C.albicans yesats. As control, cytokines produced by HSPCs without stimuli (control secretome) were measured. Cytokines whose signal intensities were statistically different, as compared with control secretome, are indicated with rectangles in the image. (B) Array Legend depicted in panel.
